# Supplementary material for: An equitable redistribution of unburnable carbon
Source: Nat Commun. 2020 Aug 7;11:3968. doi: 10.1038/s41467-020-17679-3 (PMC7414863; doi:10.1038/s41467-020-17679-3)
Supplement: Supplementary file 1 — Supplementary Information [file 41467_2020_17679_MOESM1_ESM.pdf]

Supplementary Information

An equitable redistribution of unburnable carbon

Pye et al.

Supplementary Figures

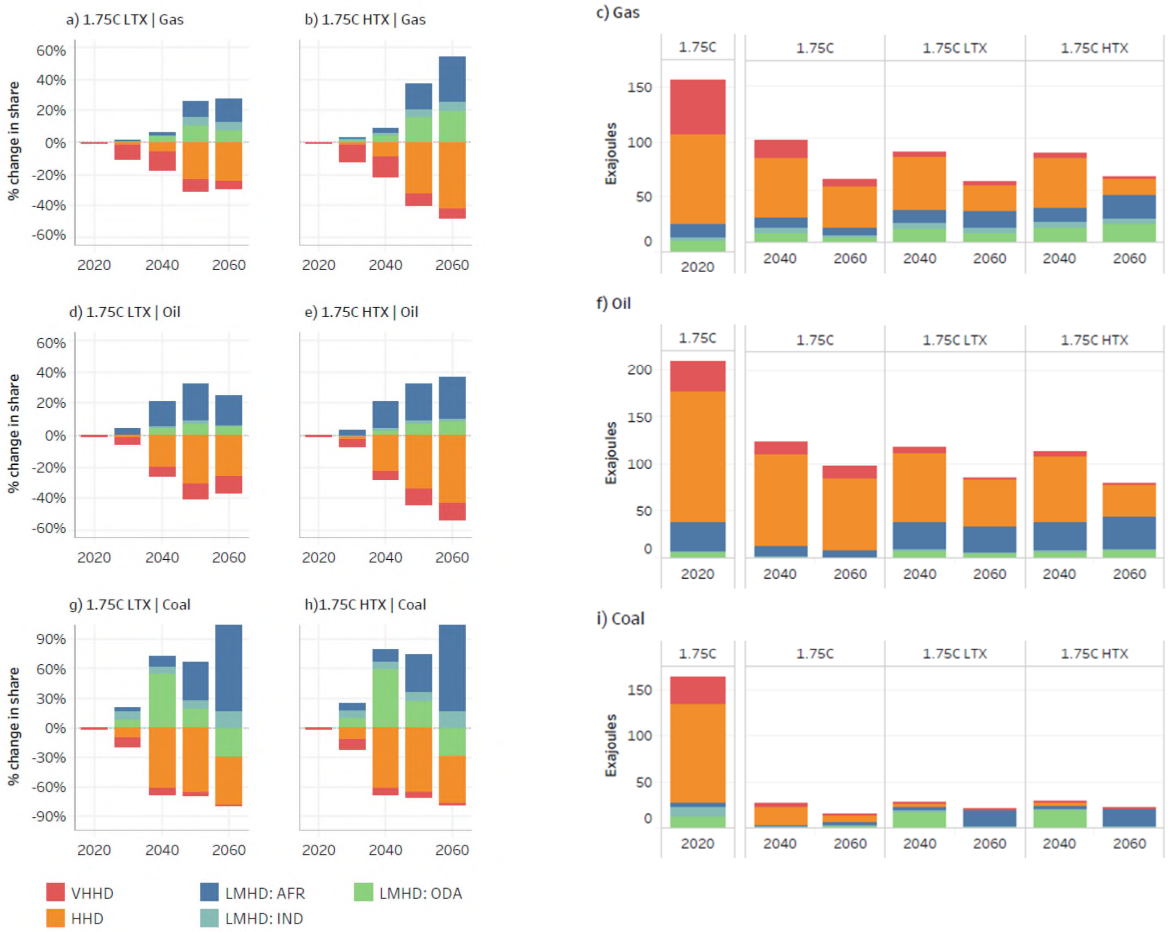

Supplementary Figure 1. Change in production levels of fossil fuels under 1.75°C HDI-based redistributed cases relative to the cost-optimal case, 2020-2060. Figures a-b for gas, d-e for oil, and g-h for coal (left-hand panel) show the annual % change in production for a region relative to total global production under the cost-optimal case. A positive change reflects a gain in total production, where a value of 20% reflects a 20% increase in share of global production observed in the optimal case. Figures c (gas), f (oil) and i (coal) in the right hand panel show the absolute levels of production, to put the percentage gains (left hand panel) in context. Source data are provided as a Source Data file.

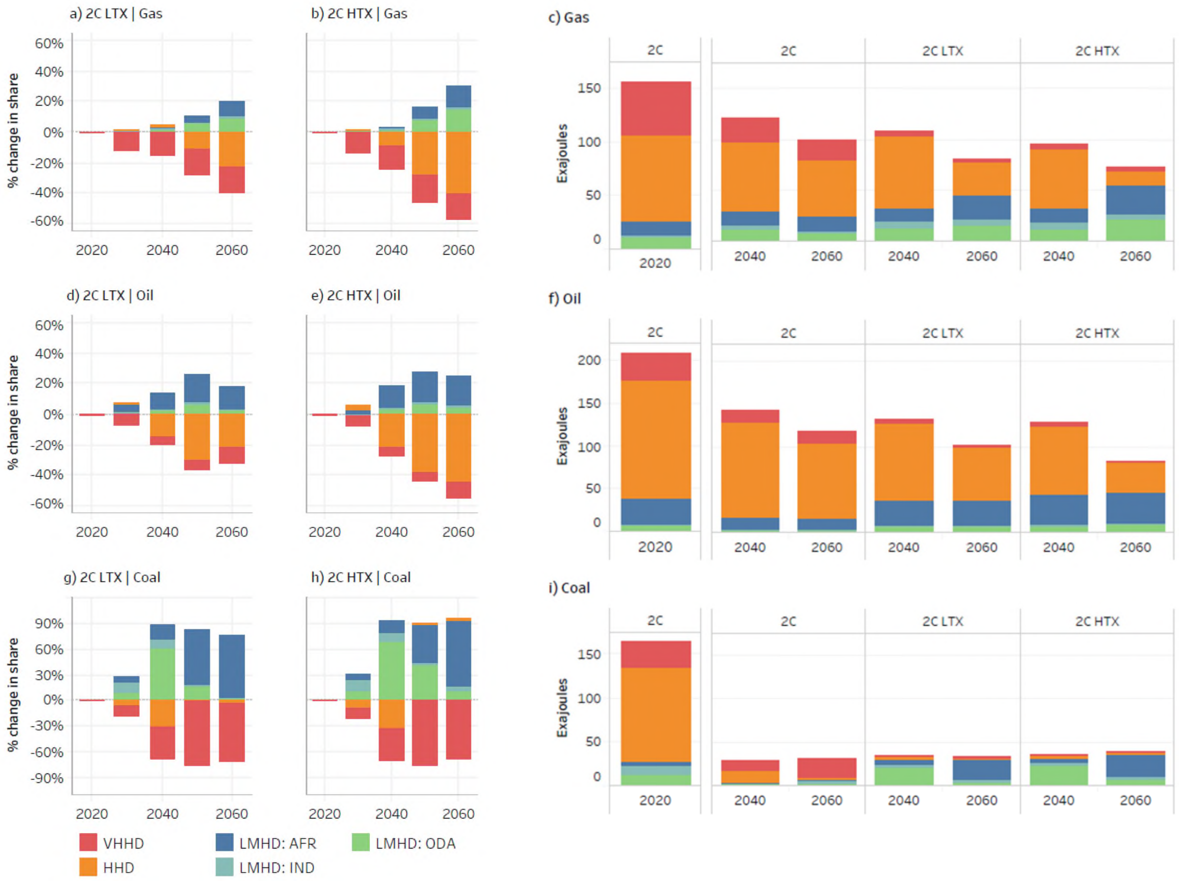

Supplementary Figure 2. Change in production levels of fossil fuels under 2°C HDI-based redistributed cases relative to the optimal case, 2020-2060. Figures a-b for gas, d-e for oil, and g-h for coal (left-hand panel) show the annual % change in production for a region relative to total global production under the cost-optimal case. A positive change reflects a gain in total production, where a value of 20% reflects a 20% increase in share of global production observed in the optimal case. Figures c (gas), f (oil) and i (coal) in the right hand panel show the absolute levels of production, to put the percentage gains (left hand panel) in context. Source data are provided as a Source Data file.

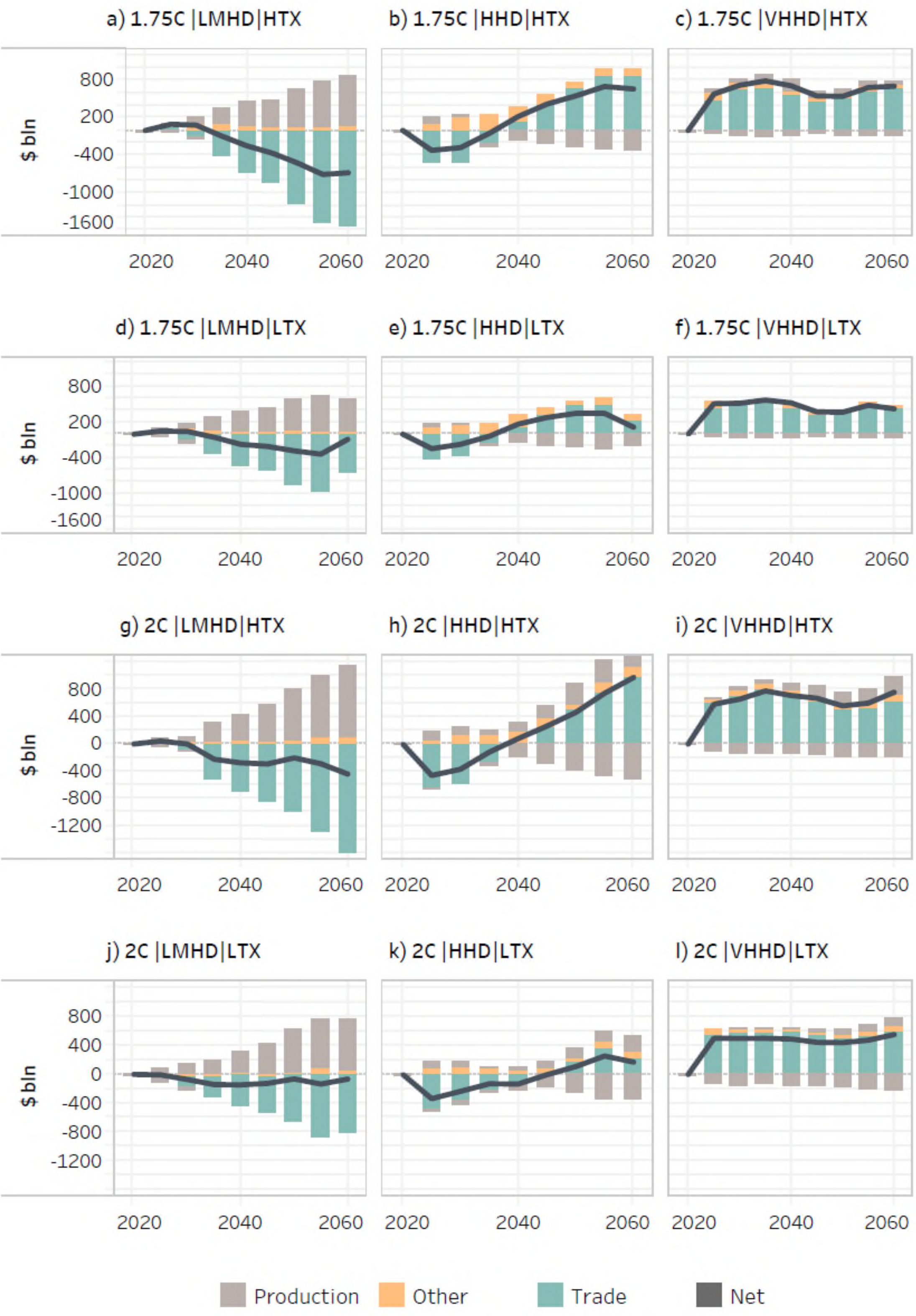

Supplementary Figure 3. Change in system cost type under high and low tax HDI-based redistributed cases relative to cost-optimal cases by HDI region, 2020-2060. Figures represent the change in energy system costs by cost type for each HDI group, compared to the cost-optimal case. Cost categories are as follows: Production (including investment and O&M costs), trade (balance of import-export costs), and other (consumer surplus losses associated with demand response). Negative values show a reduction in costs, while the black trend line shows the net change in total costs. The upper panel (a-f) is for the 1.75°C cases and lower panel (g-l) for the 2°C cases. Source data are provided as a Source Data file.

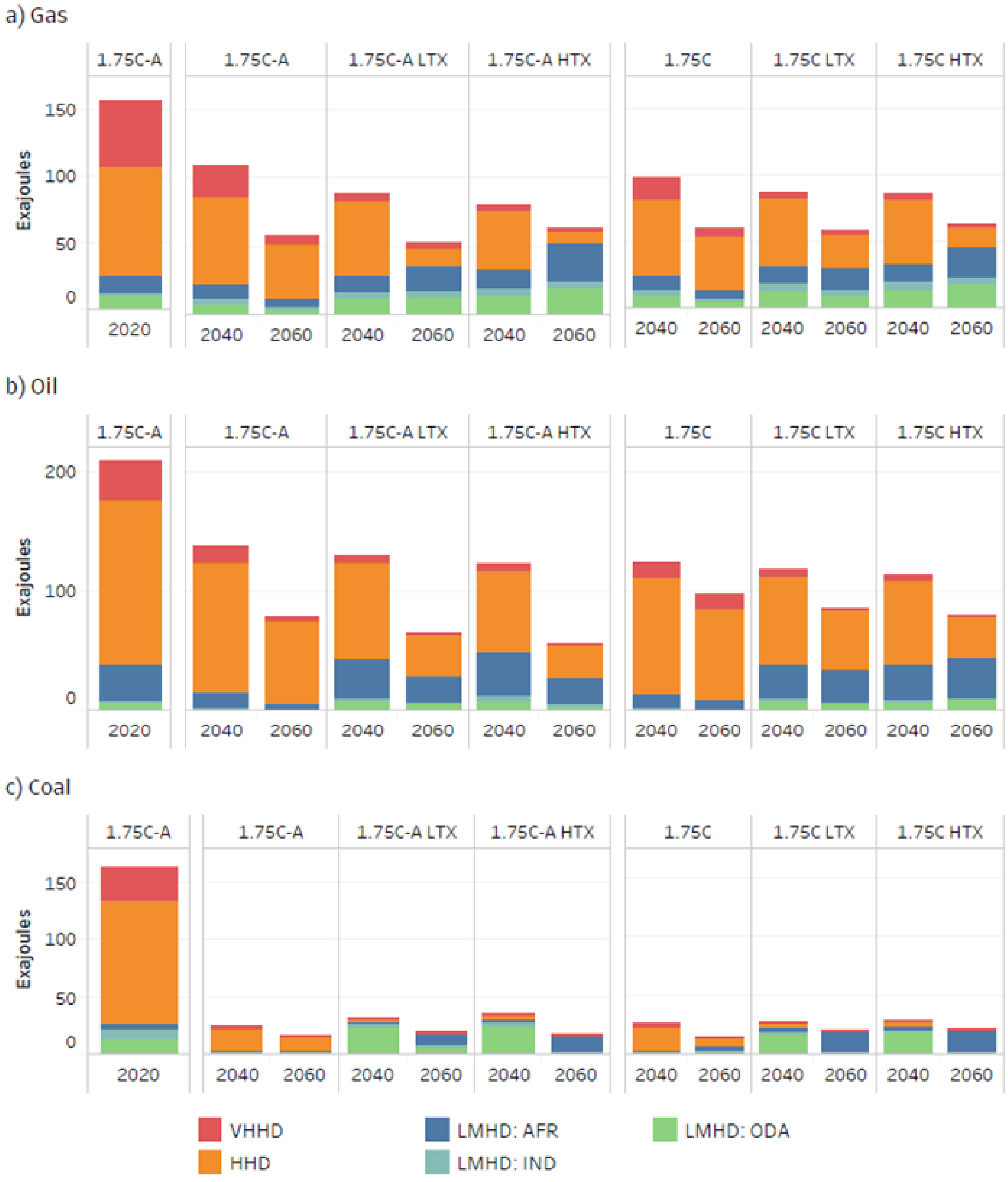

Supplementary Figure 4. Production levels of fossil fuels under 1.75°C redistributed cases under HDI criteria, under SSP2 and SSP1 demand drivers, 2020-2060. Figures on the left, using scenario name '1.75C-A' show the absolute levels of production under SSP1 demand, and on the right, under SSP2 demands. The 2020 value is the same for all scenarios. Source data are provided as a Source Data file.

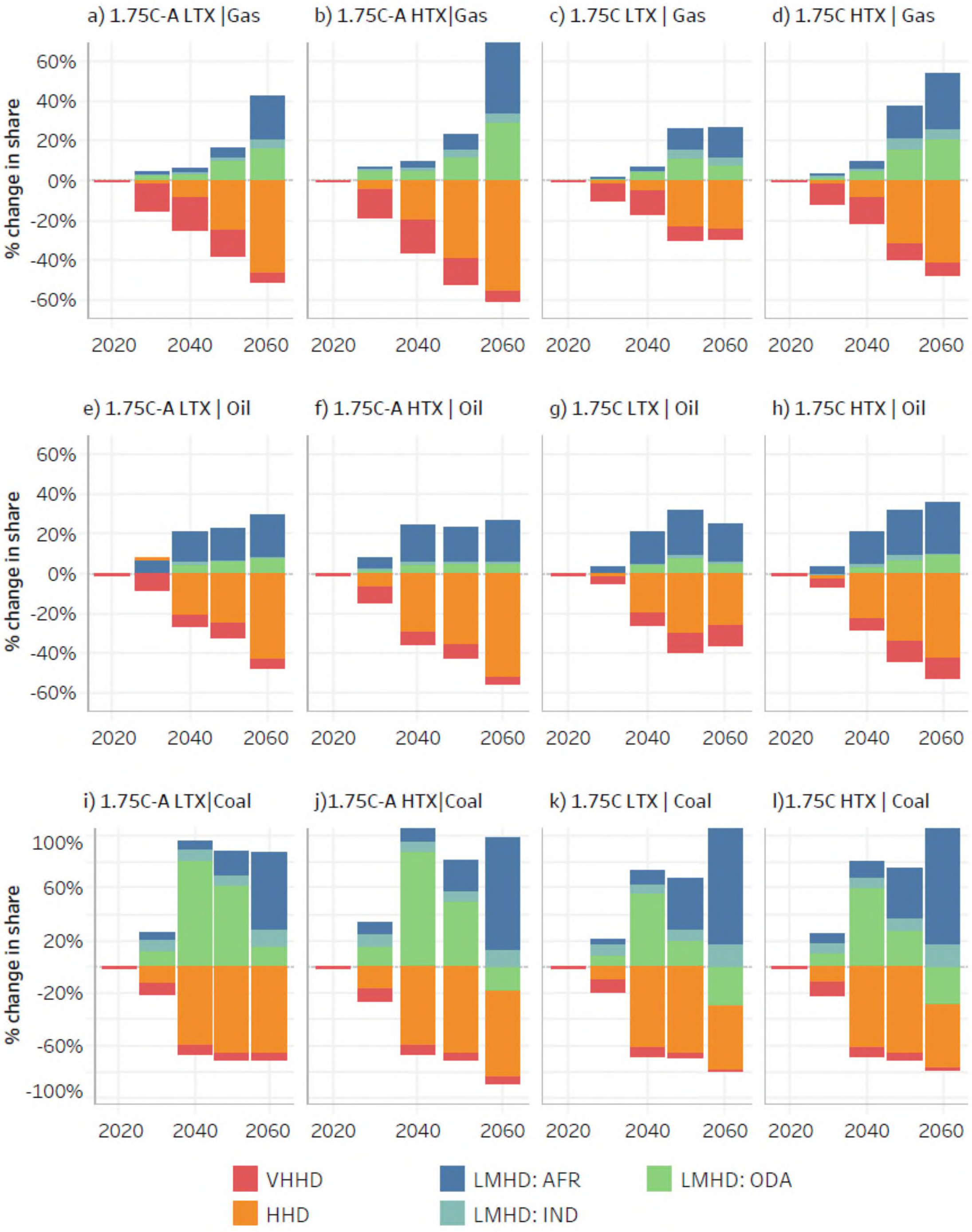

Supplementary Figure 5. Change in production levels of fossil fuels under 1.75°C HDI redistributed cases relative to the optimal case, 2020-2060. Figures in the left-hand panel show the annual % change in production for a region under SSP1 demand based on the HDI criteria, relative to total global production under the cost-optimal case (1.75C-A). A positive change reflects a gain in total production, where a value of 20% reflects a 20% increase in share of global production observed in the optimal case. The right-hand panel shows the equivalent results under the HDI criteria based on SSP2 demands. Source data are provided as a Source Data file.

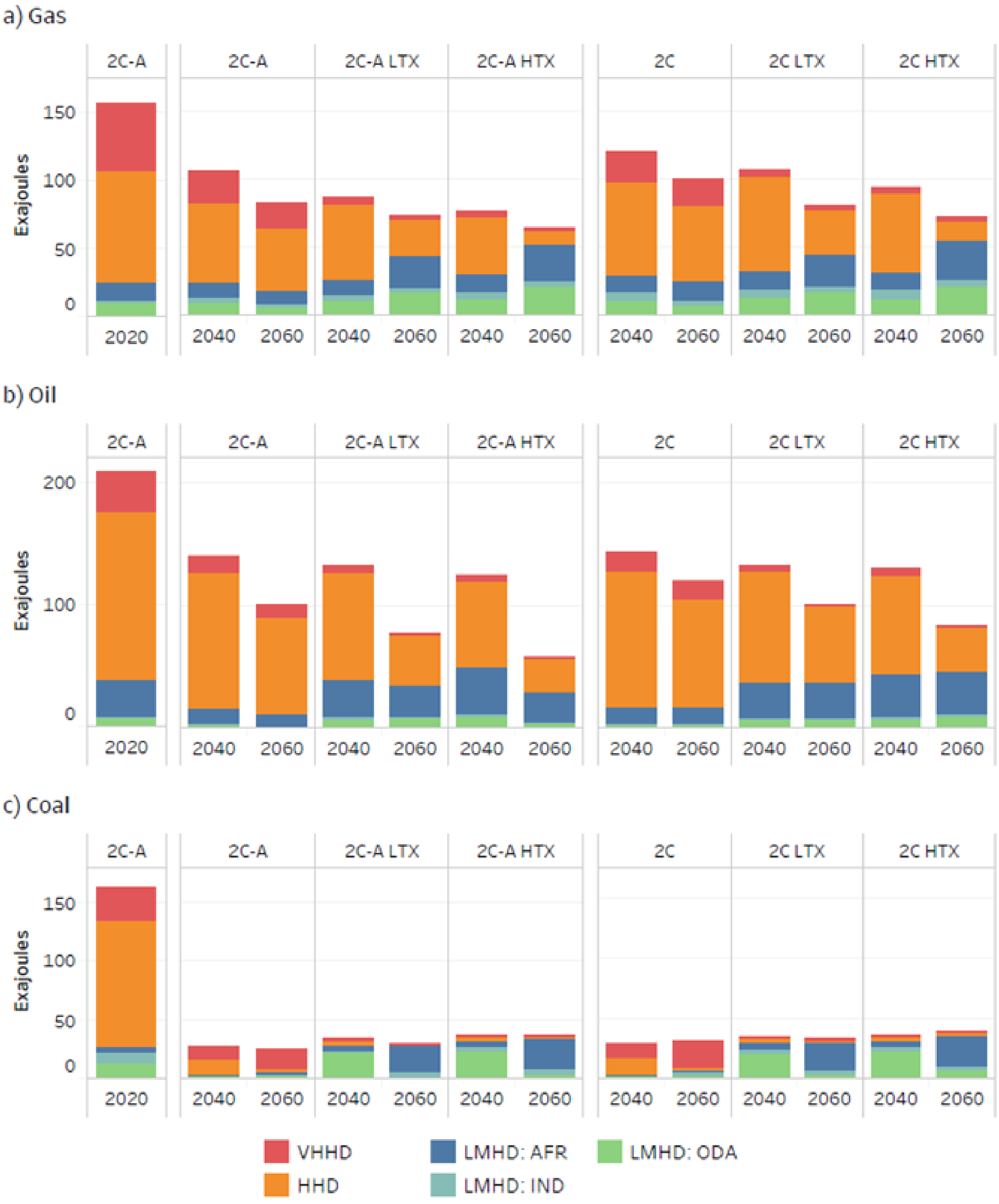

Supplementary Figure 6. Production levels of fossil fuels under 2°C redistributed cases under HDI criteria, under SSP2 and SSP1 demand drivers, 2020-2060. Figures on the left, using scenario name '2C-A' show the absolute levels of production under SSP1 demand, and on the right, under SSP2 demands. The 2020 value is the same for all scenarios. Source data are provided as a Source Data file.

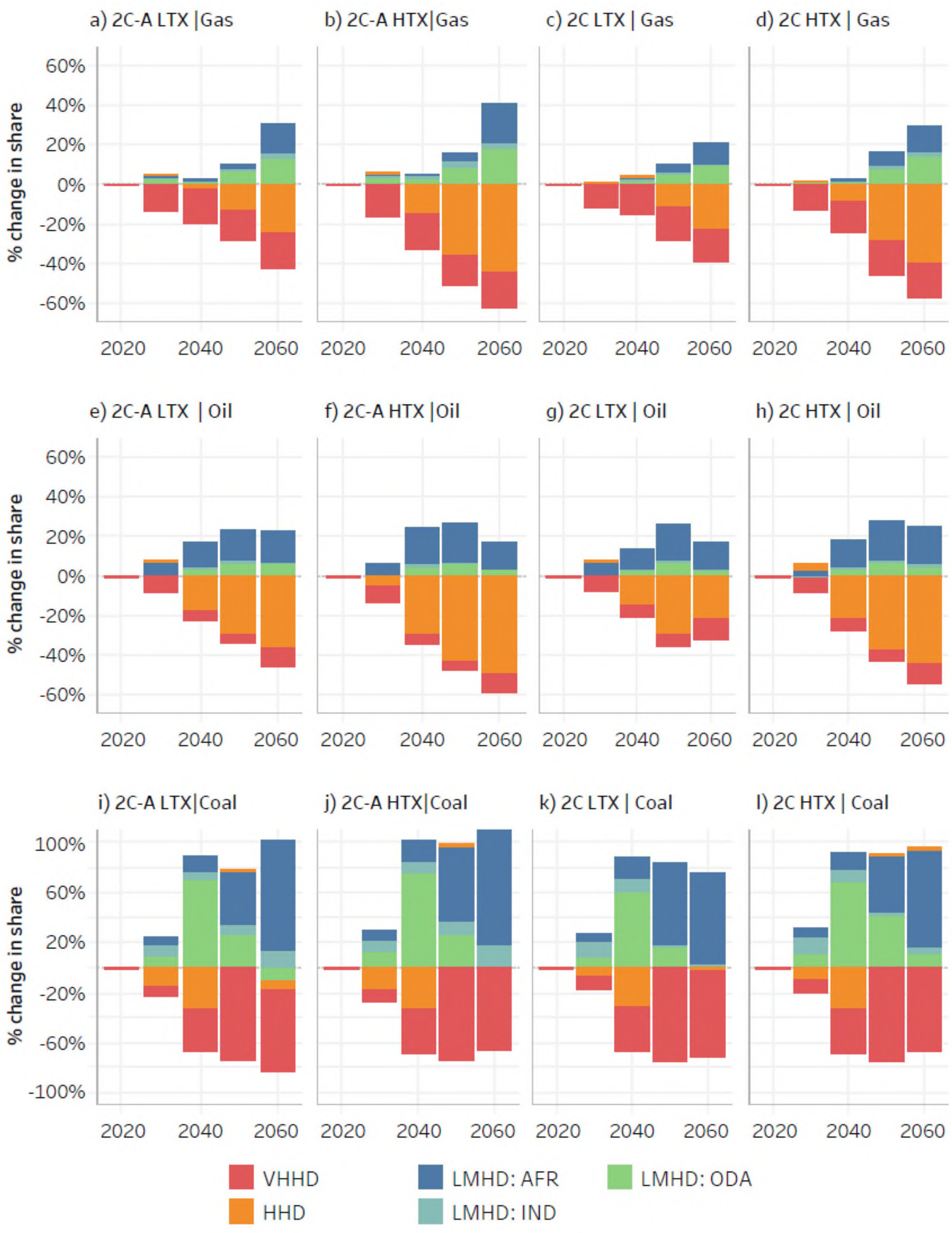

Supplementary Figure 7. Change in production levels of fossil fuels under 2°C HDI redistributed cases relative to the optimal case, 2020-2060. Figures in the left-hand panel show the annual % change in production for a region under SSP1 demand based on the HDI criteria (2C-A), relative to total global production under the cost-optimal case. A positive change reflects a gain in total production, where a value of 20% reflects a 20% increase in share of global production observed in the optimal case. The right-hand panel shows the equivalent results under the HDI criteria based on SSP2 demands. Source data are provided as a Source Data file.

i)

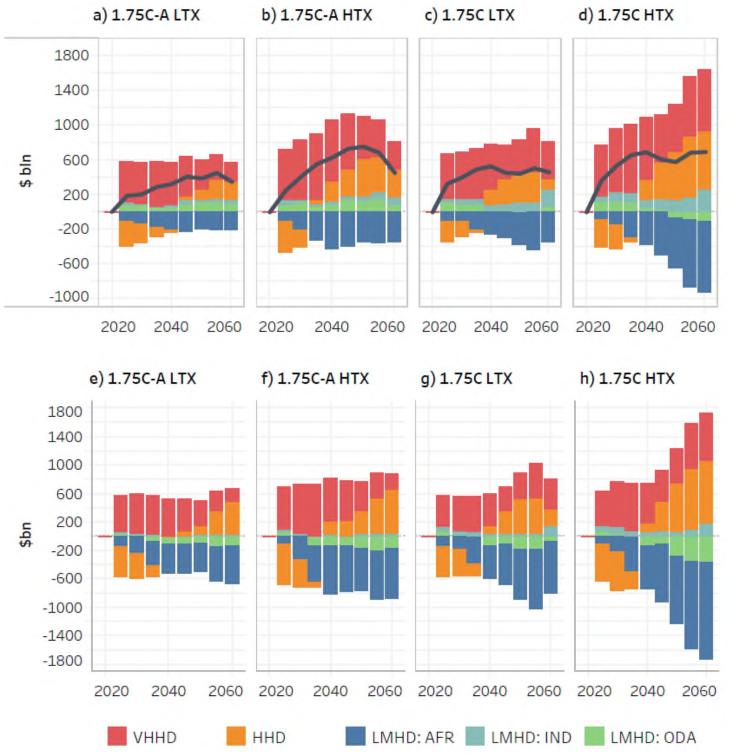

ii)

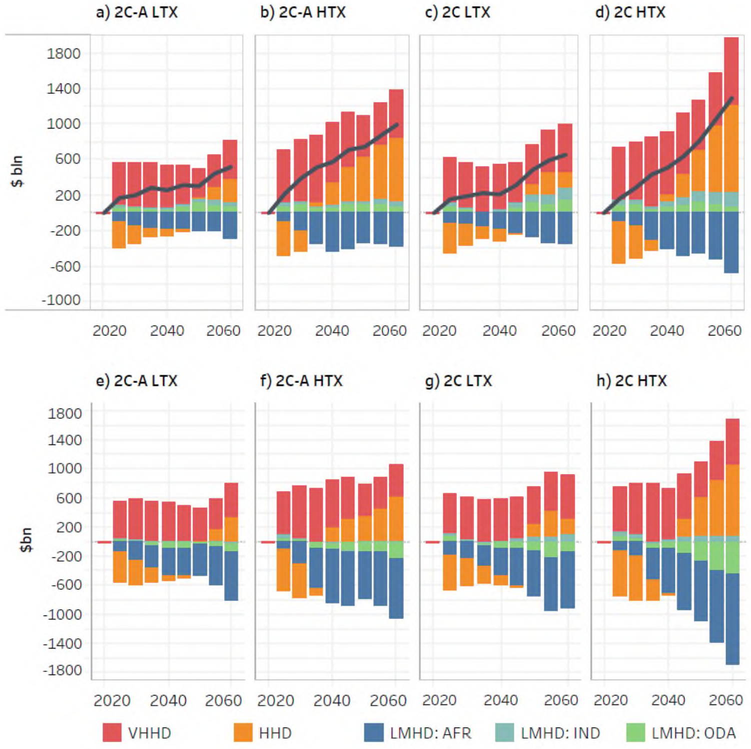

Supplementary Figure 8. Change in system costs and regional commodity trade costs under i) 1.75°C and ii) 2°C HDI-based redistributed cases relative to cost-optimal cases, 2020-2060. The left hand graphs represent SSP1 scenarios while right hand graphs represent SSP2 scenarios. Figures in the upper panel (a-d) represent the change in energy system costs by equity-based group, compared to the cost-optimal case. Negative values show a reduction in costs, while the black trend line shows the net change in global costs. Figures in the lower panel (e-h) show the change in commodity trade costs by equity-based group, compared to the cost-optimal case, reflecting the balance between export revenues and import costs. Negative values show an increase in net exports. Source data are provided as a Source Data file.

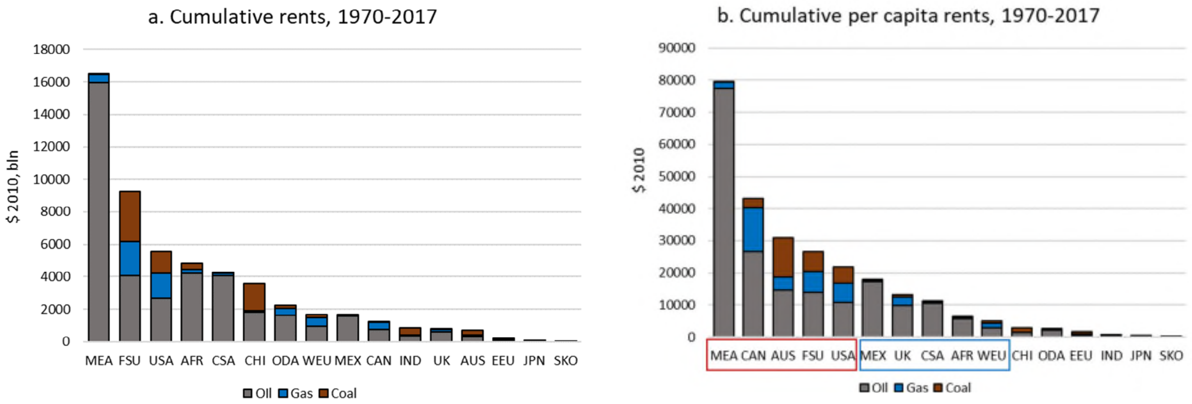

Supplementary Figure 9. Cumulative rents (a) and cumulative per capita rents (b) by TIAM-UCL model region, 1970-2017. Countries included in the regional grouping are listed in Supplementary Table 18. Source data are provided as a Source Data file.

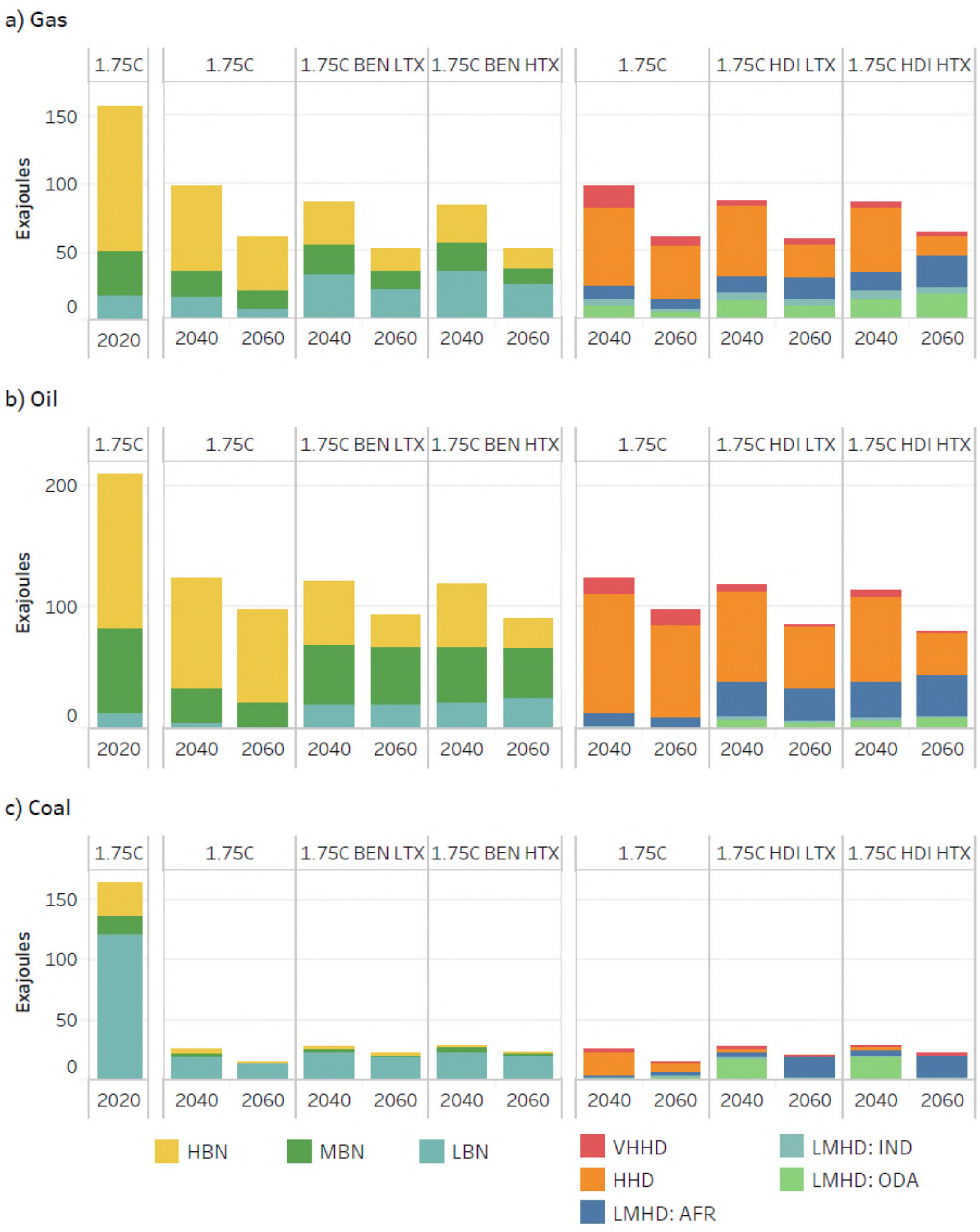

Supplementary Figure 10. Production levels of fossil fuels under 1.75°C redistributed cases under both equity criteria, 2020-2060. Figures on the left show the absolute levels of production under the 'accrued benefits' criteria, and on the right, under the HDI criteria. Source data are provided as a Source Data file.

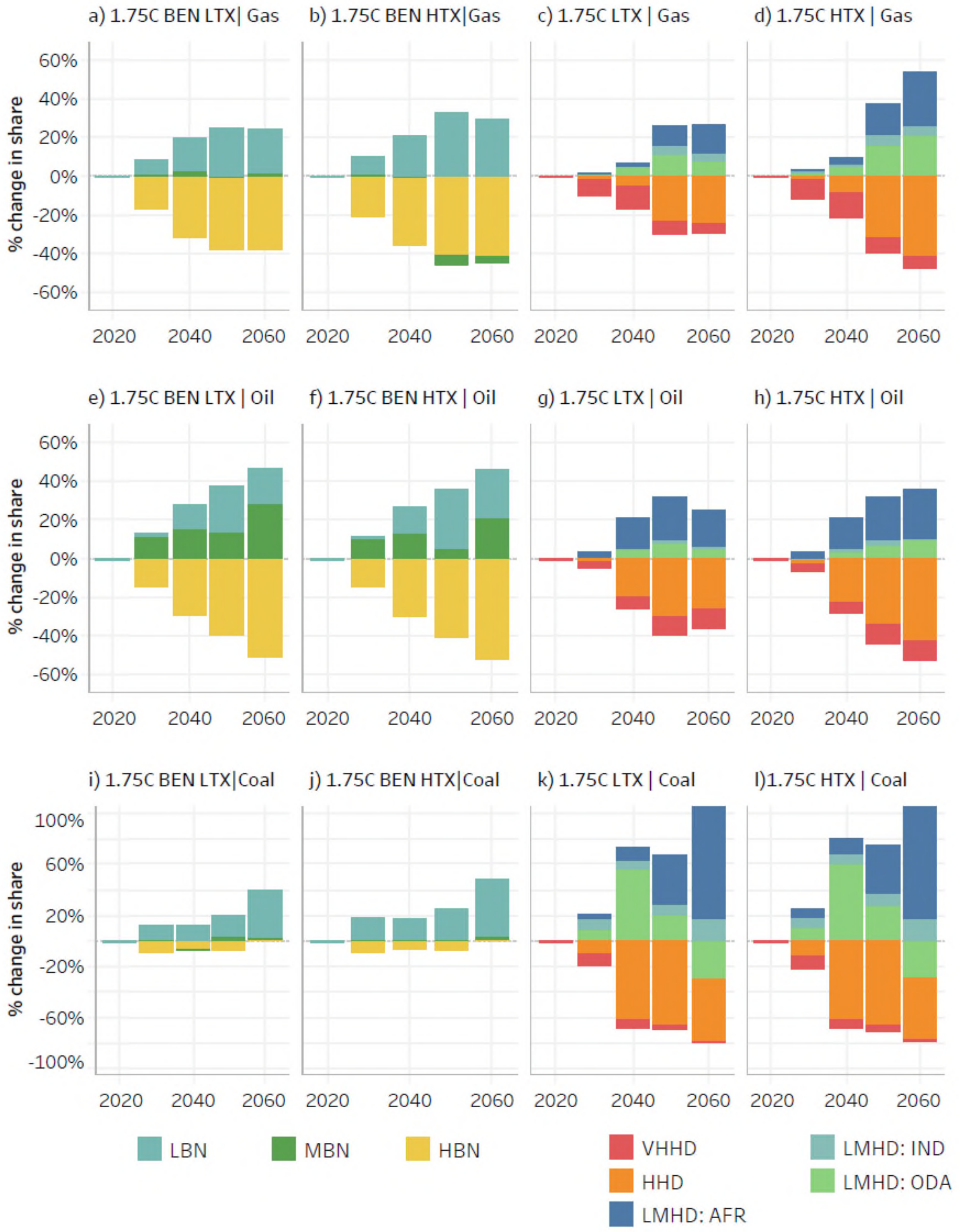

Supplementary Figure 11. Change in production levels of fossil fuels under 1.75°C redistributed cases under both equity criteria relative to the optimal case, 2020-2060. Figures in the left-hand panel (yellow-green-blue shades) show the annual % change in production for a region under the accrued benefit criteria, relative to total global production under the cost-optimal case. A positive change reflects a gain in total production, where a value of 20% reflects a 20% increase in share of global production observed in the optimal case. The right-hand panel shows the equivalent results under the HDI criteria. Source data are provided as a Source Data file.

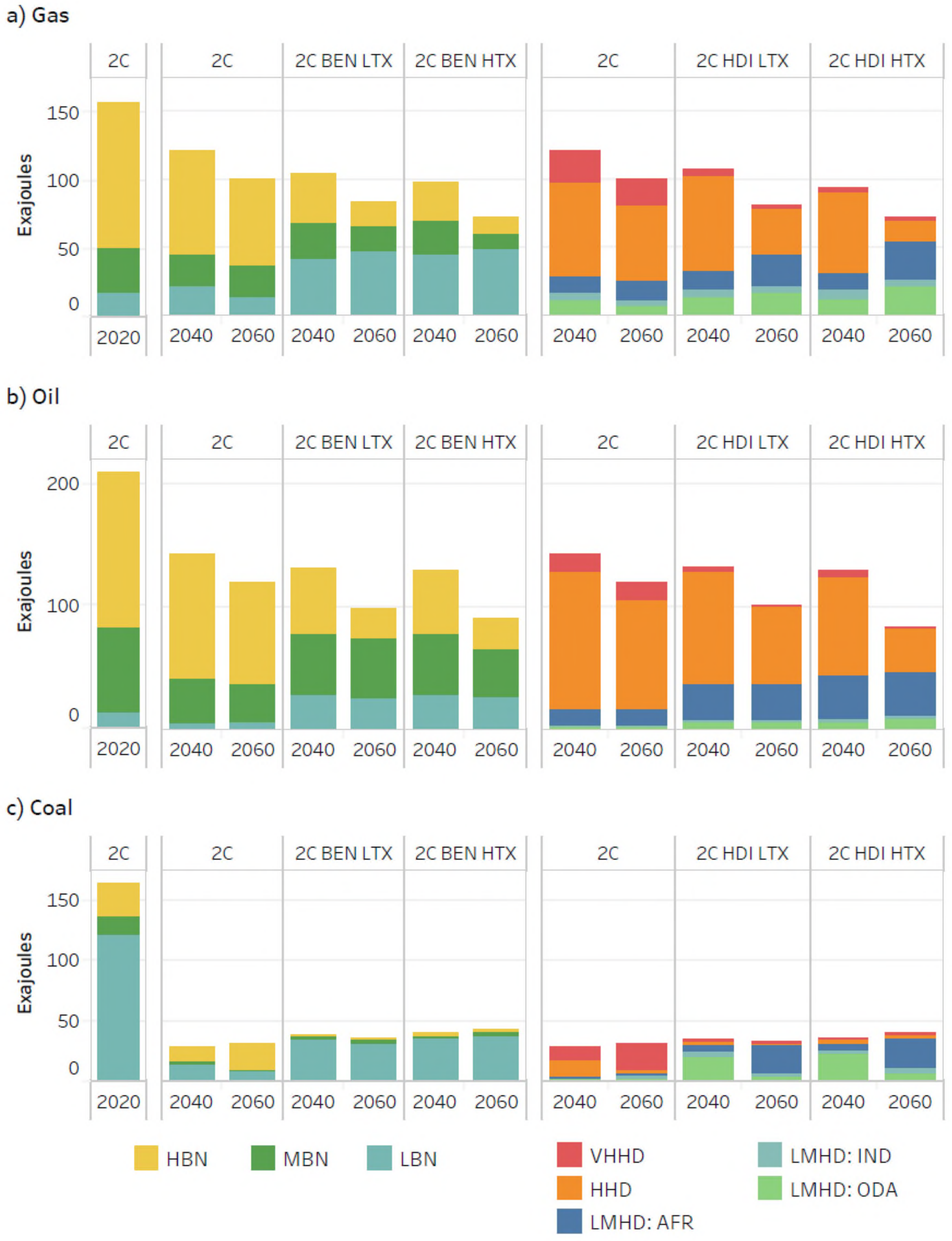

Supplementary Figure 12. Change in production levels of fossil fuels under 2°C redistributed cases under both equity criteria, 2020-2060. Figures on the left show the absolute levels of production under the 'accrued benefits' criteria, and on the right, under the HDI criteria. Source data are provided as a Source Data file.

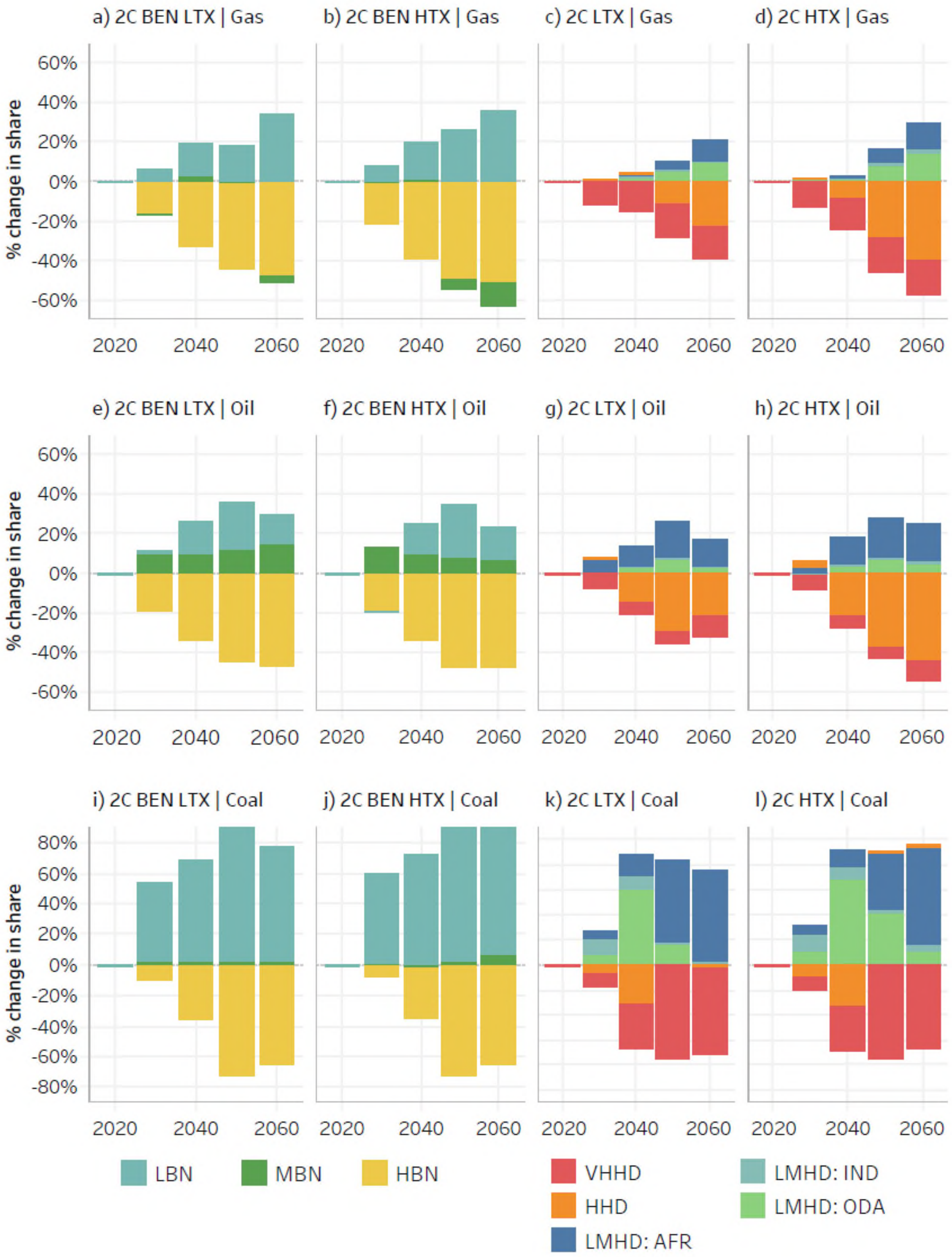

Supplementary Figure 13. Change in production levels of fossil fuels under 2°C redistributed cases under both equity criteria relative to the optimal case, 2020-2060. Figures in the left-hand panel (yellow-green-blue shades) show the annual % change in production for a region under the accrued benefit criteria, relative to total global production under the cost-optimal case. A positive change reflects a gain in total production, where a value of 20% reflects a 20% increase in share of global production observed in the optimal case. The right-hand panel shows the equivalent results under the HDI criteria. Source data are provided as a Source Data file.

i)

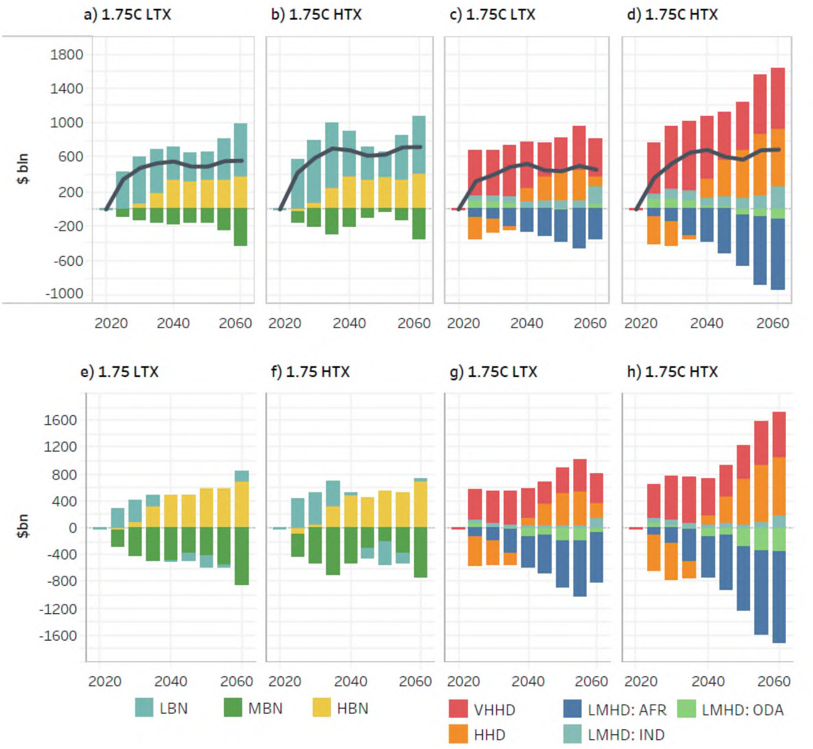

ii)

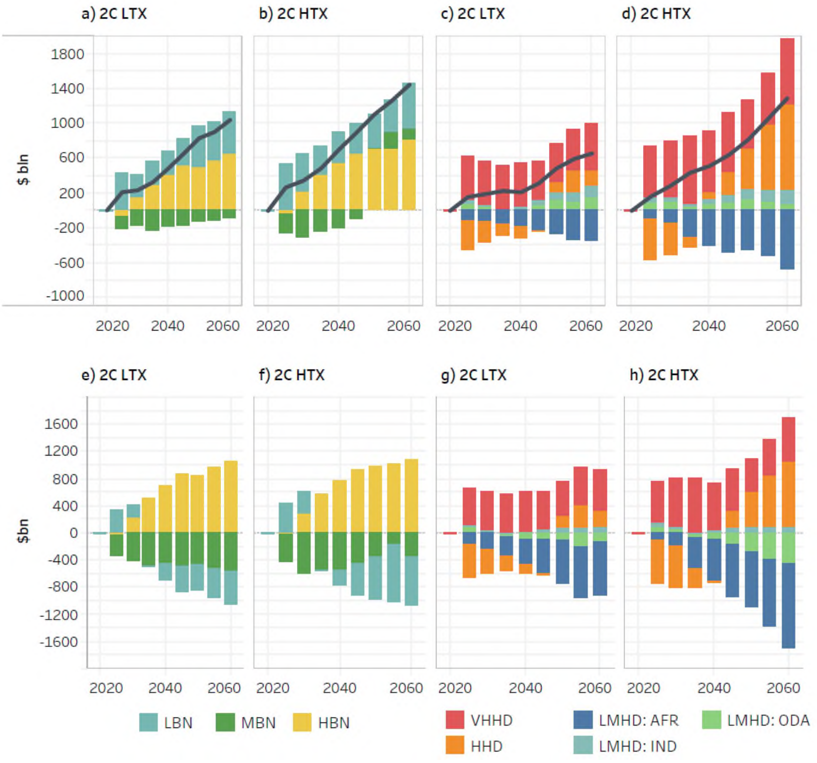

Supplementary Figure 14. Change in system costs and regional commodity trade costs under i) 1.75°C and ii) 2°C redistributed cases relative to cost-optimal cases, 2020-2060. Figures in the upper panel (a-d) represent the change in energy system costs by equity-based group, compared to the cost-optimal case. Negative values show a reduction in costs, while the black trend line shows the net change in global costs. Figures in the lower panel (e-h) show the change in commodity trade costs by equity-based group, compared to the cost-optimal case, reflecting the balance between export revenues and import costs. Negative values show an increase in net exports. Source data are provided as a Source Data file.

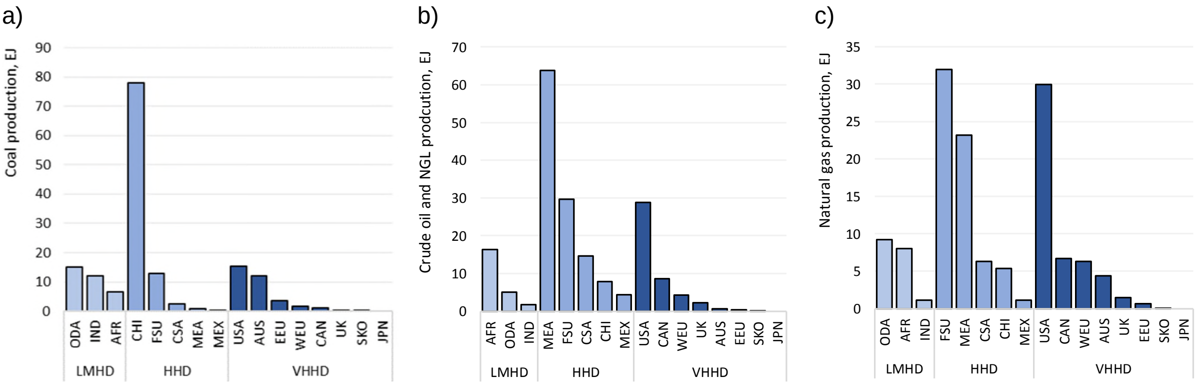

Supplementary Figure 15. Fossil fuel production by model region and HDI grouping, 2018 (Source: IEA, 2019[1]). a. Coal production; b) Oil production (crude and NGL); and c. Natural gas production. See Supplementary Table 18 for region name abbreviations. Source data are provided as a Source Data file.

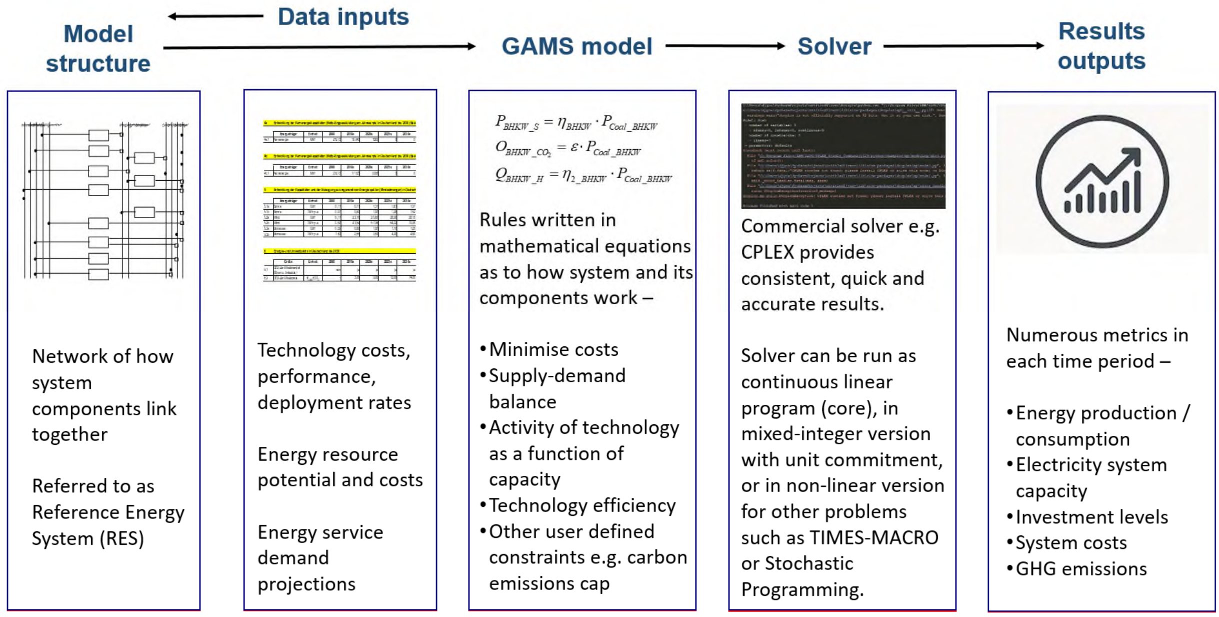

Supplementary Figure 16. Core components of the TIMES model framework

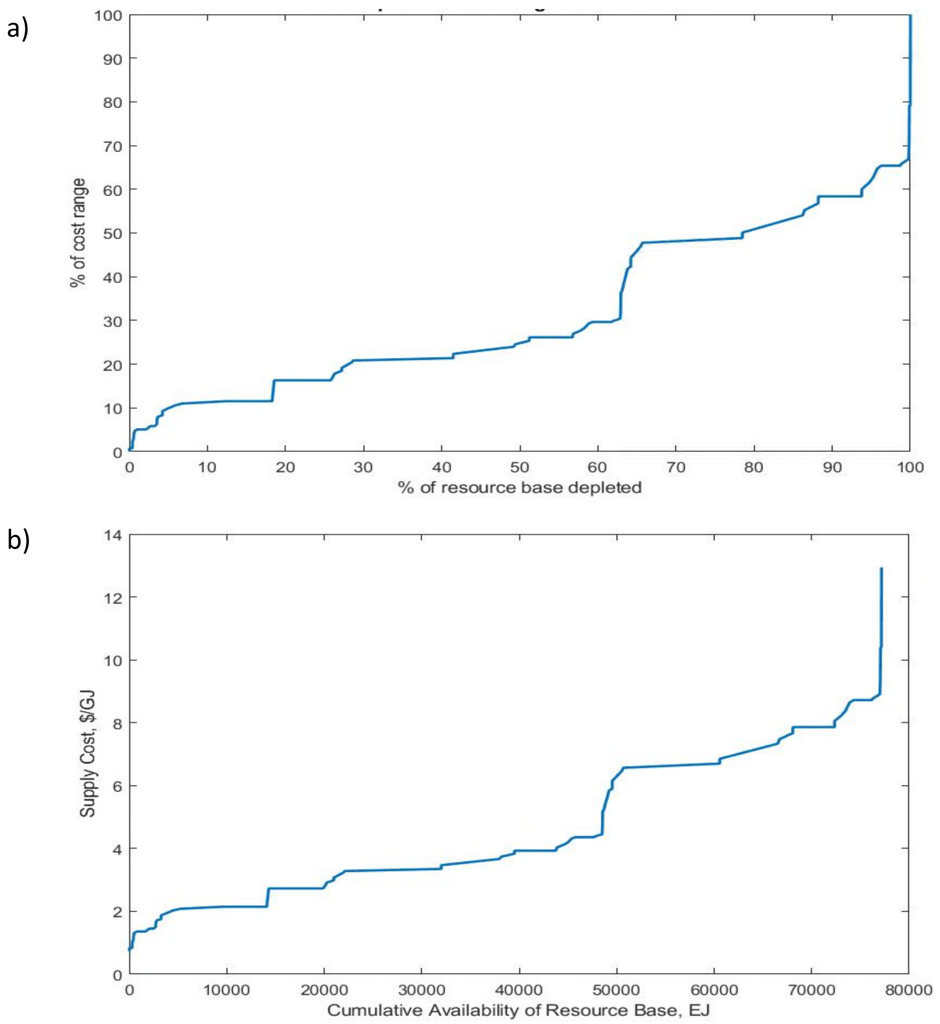

Supplementary Figure 17. a) Cost depletion curve derived from TIAM-UCL resources and costs for global coal; b) supply cost curve derived from the cost depletion curve. Source data are provided as a Source Data file.

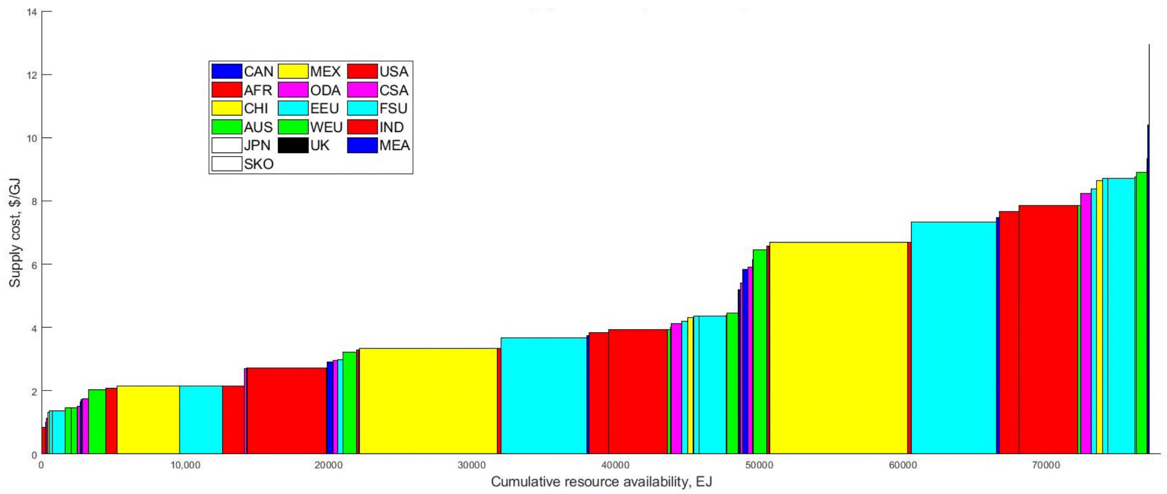

Supplementary Figure 18. Global supply cost curve for coal disaggregated into TIAM-UCL regions. Source data are provided as a Source Data file.

a)

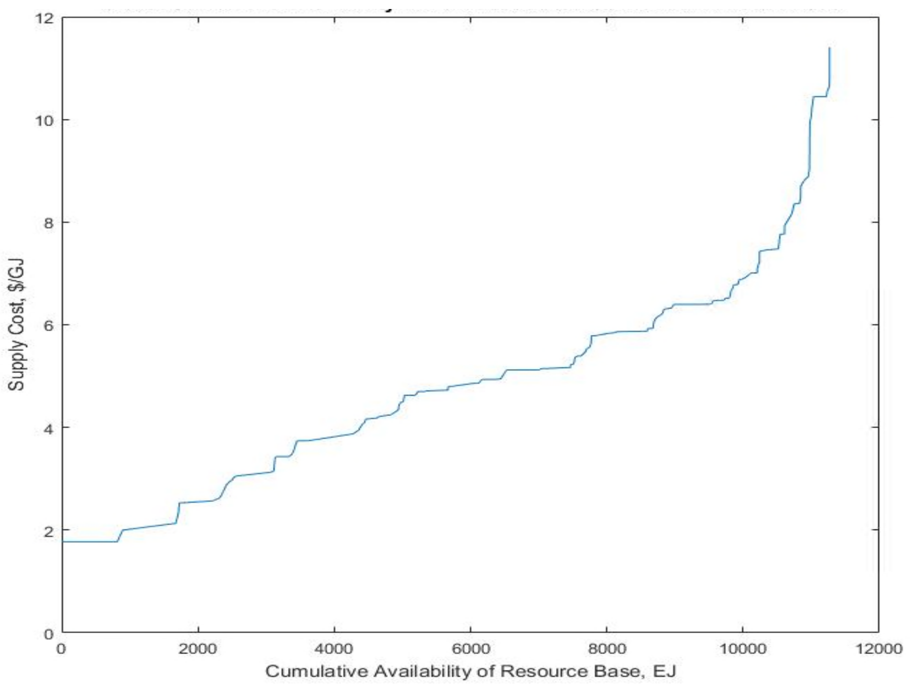

b)

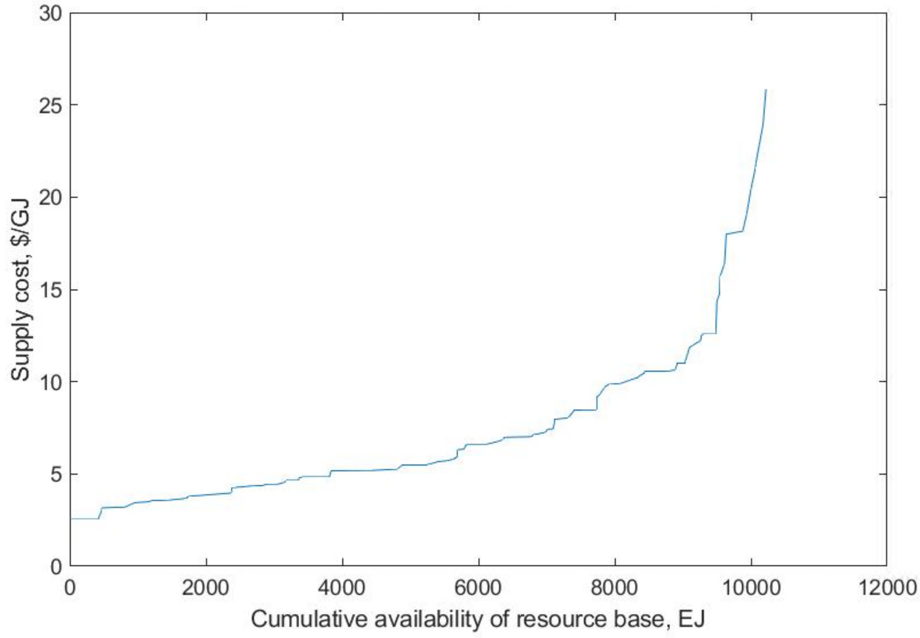

Supplementary Figure 19. Global central (P50) supply cost curve for a) non-associated conventional natural gas and b) unconventional natural gas resources. Source data are provided as a Source Data file. Source: Welsby, 2020[2]

a)

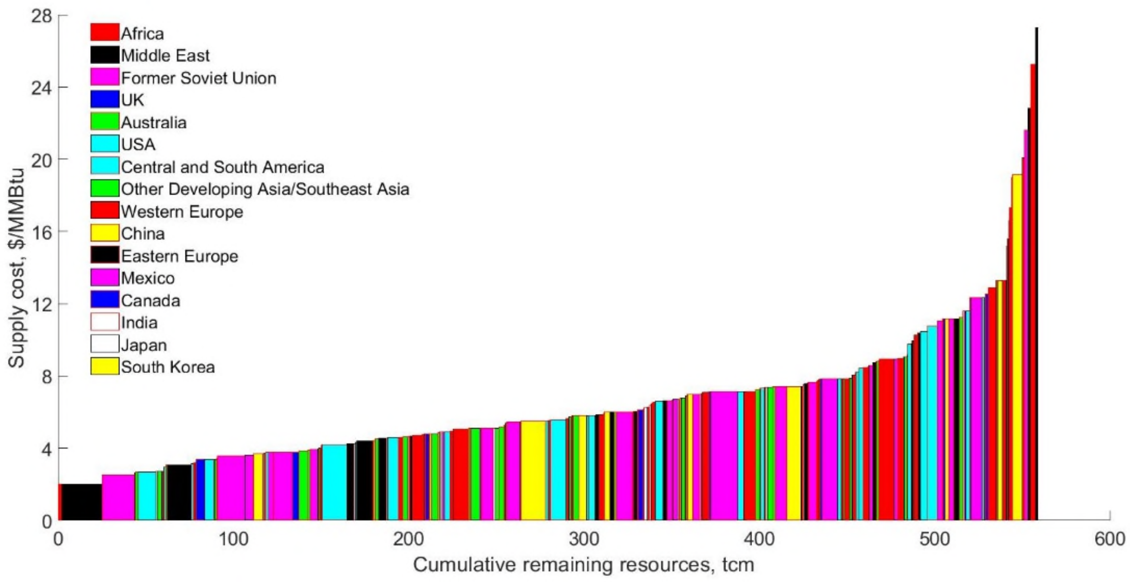

b)

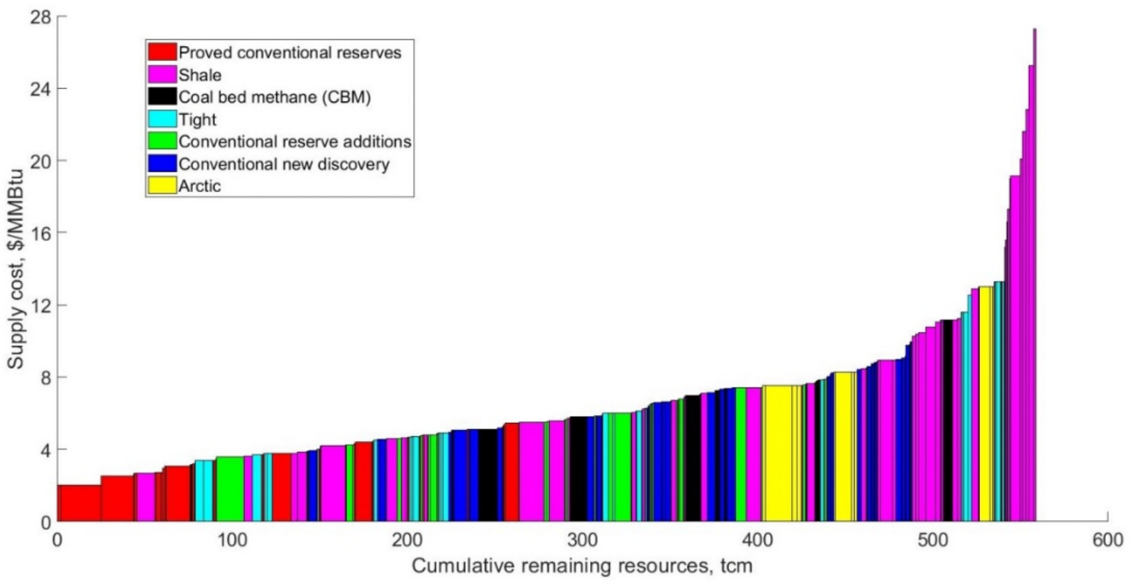

Supplementary Figure 20. Global gas supply cost curve from 2015 by a) region b) resource category. Source data are provided as a Source Data file. Source: Welsby, 2020[2], McGlade, 2013[3]

a)

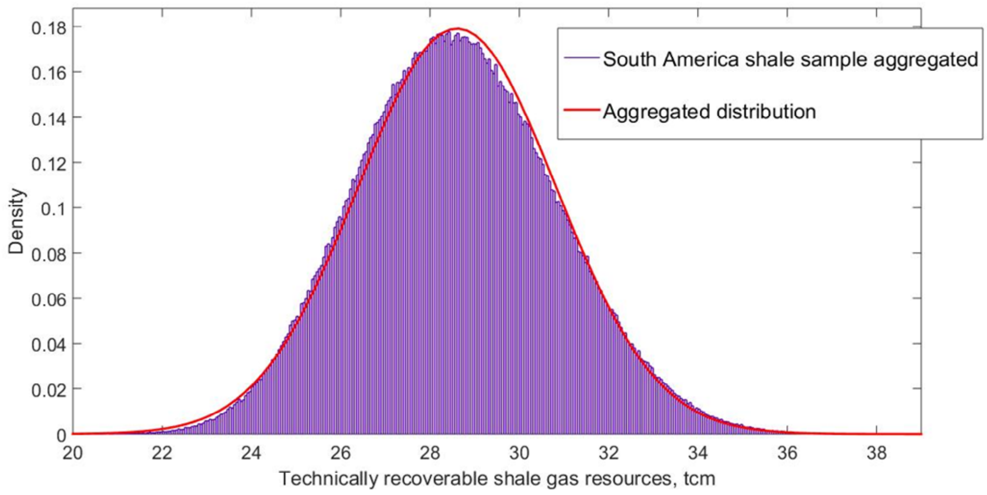

b)

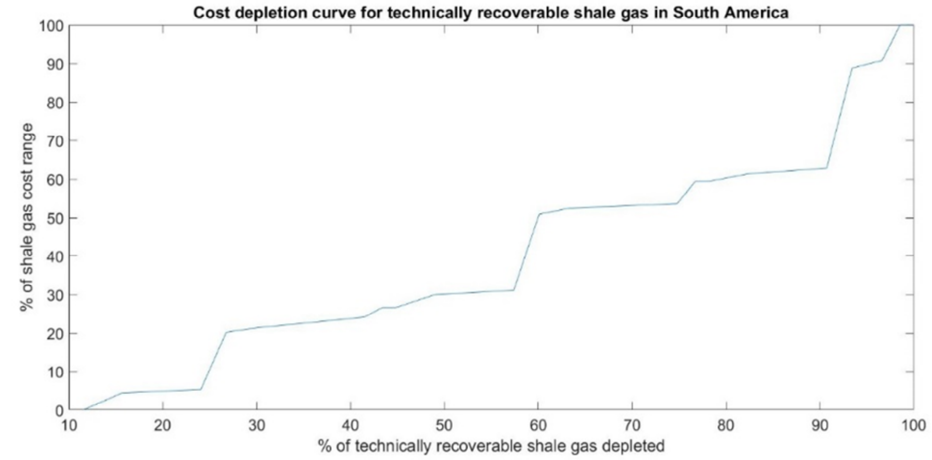

c)

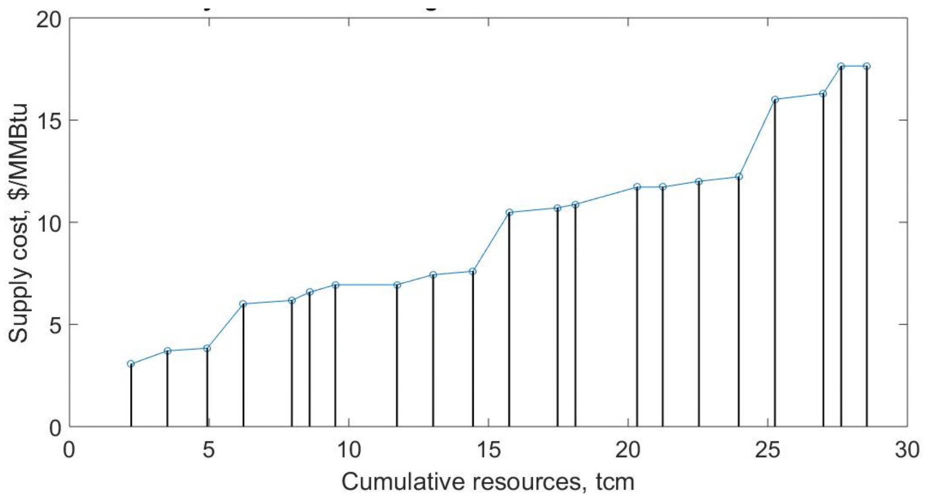

Supplementary Figure 21. a) Aggregated distribution for estimates of technically recoverable resources of shale gas in individual plays in Central and South America (CSA) region in TIAM-UCL; b) Cost depletion curve for shale gas in Central and South America, and c) central supply cost curve for technically recoverable resources of shale gas for Central and South America (CSA). Source data are provided as a Source Data file. Source: Welsby, 2020[2]

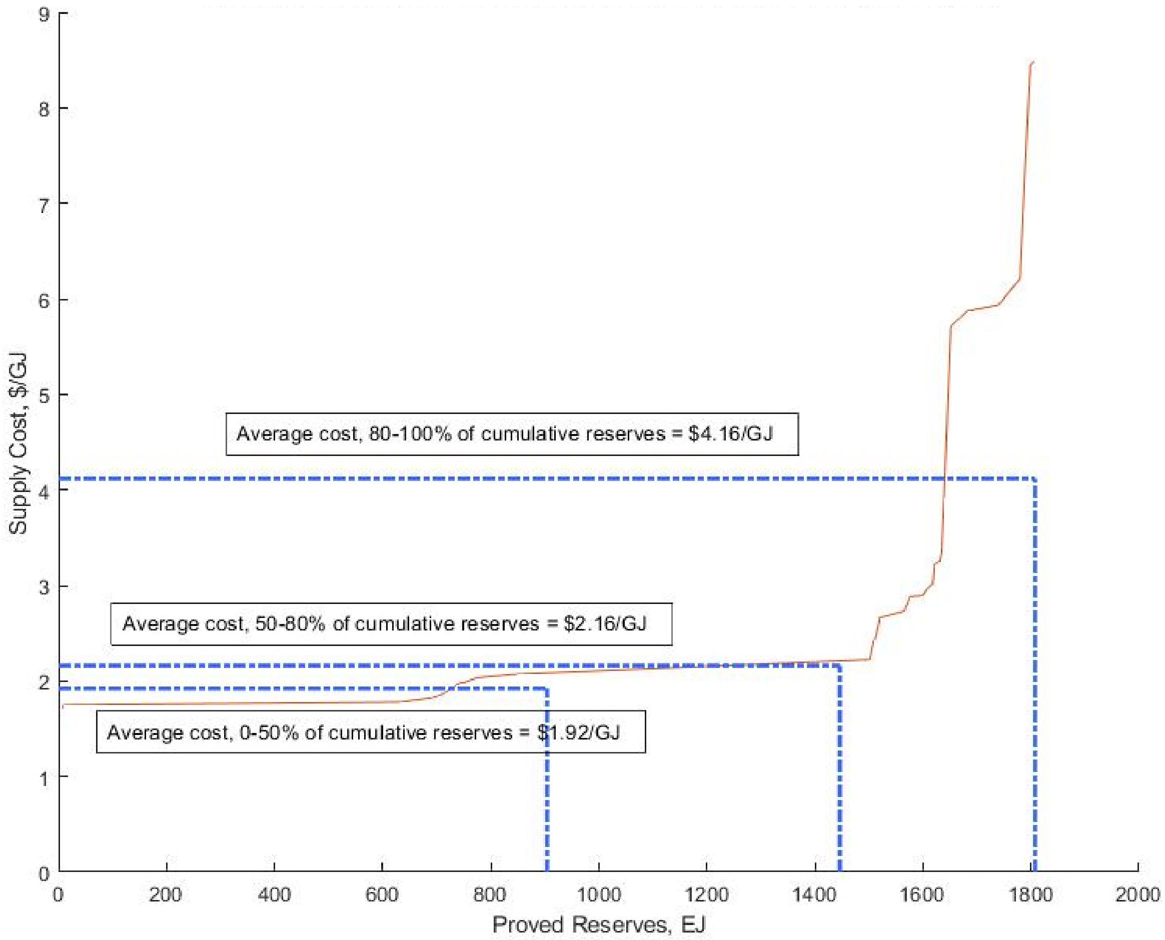

Supplementary Figure 22. Example of three-step supply cost curve for the Middle East OPEC region in TIAM-UCL, for proved non-associated conventional gas reserves. Source data are provided as a Source Data file. Source: Welsby, 2020[2]

a)

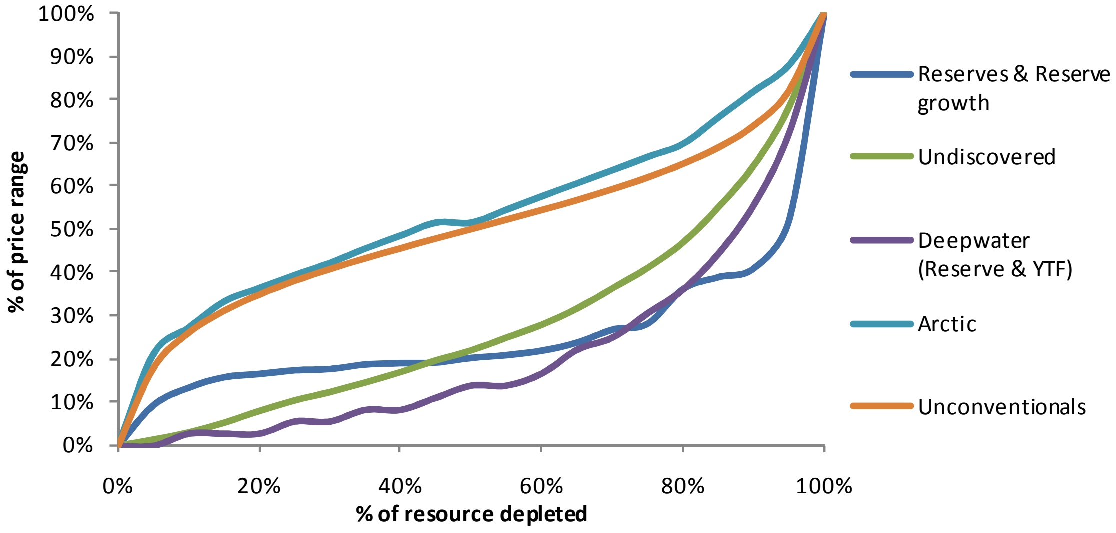

b)

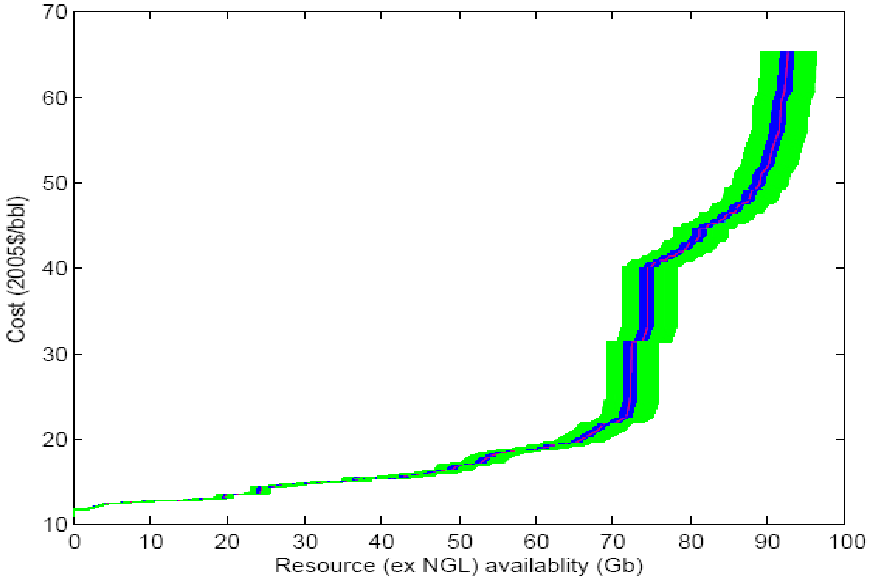

Supplementary Figure 23. a) Cost depletion curves for a range of oil categories, and b) supply cost curves for African OPEC countries showing the range of uncertainty from the output distribution, and combined with representative cost depletion curves in a), to form a range of potential reserve-cost combinations. Source: McGlade, 2013[3]

a)

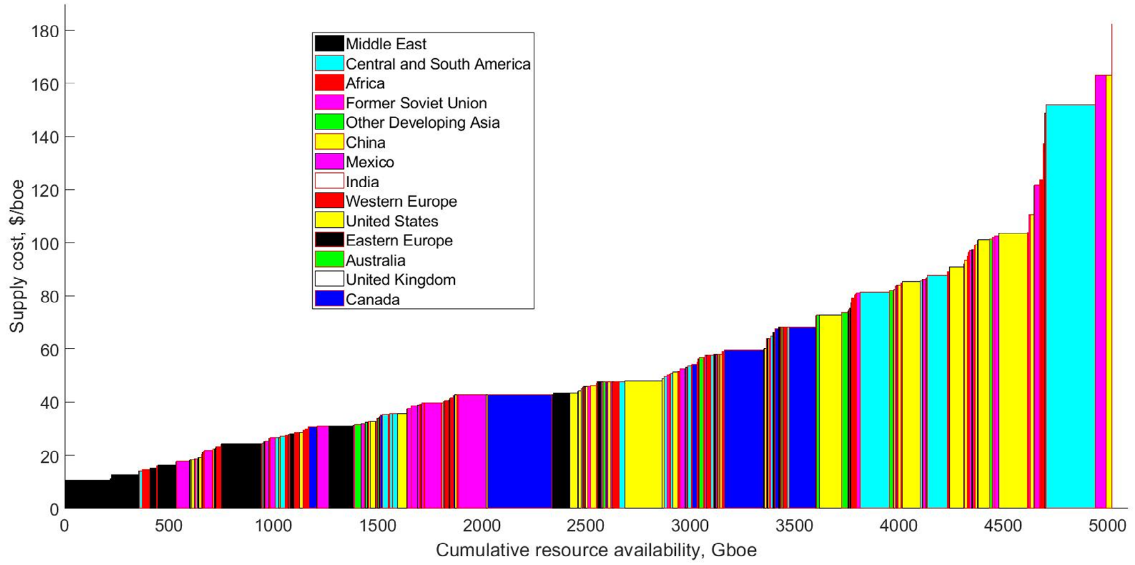

b)

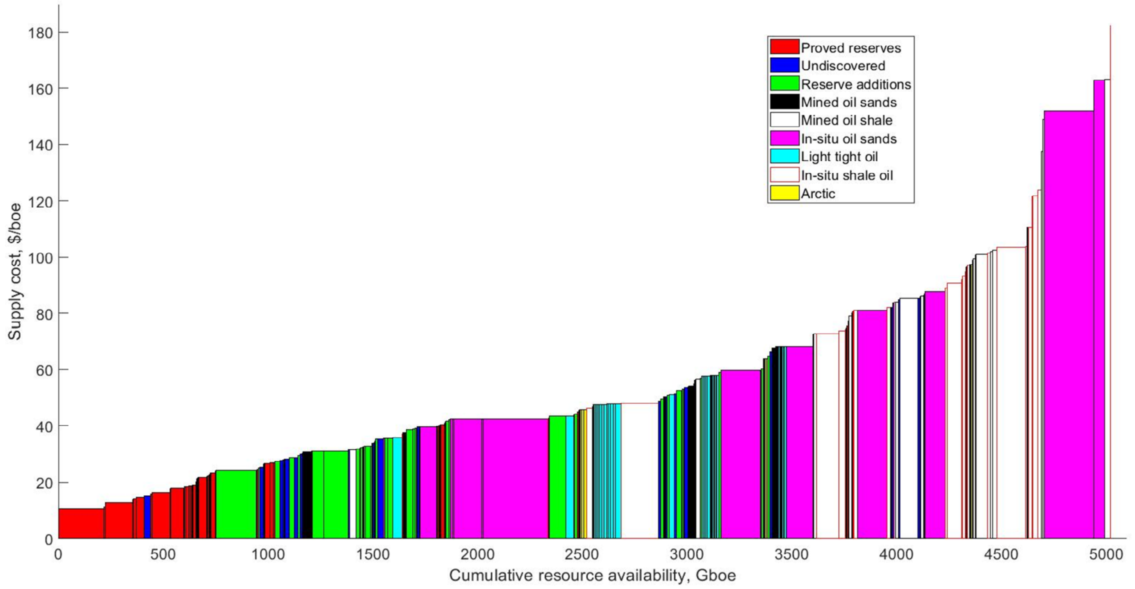

Supplementary Figure 24. Global supply cost curve for oil from 2015 by a) TIAM-UCL region and b) oil resource category. Source data are provided as a Source Data file.

a)

~TradeLinks																			
OILCRD	AFR	AUS	CAN	CHI	CSA	EEU	FSU	GBL	IND	JPN	MEA	MEX	ODA	SKO	USA	WEU	UK	MINRNW	IMPEXP
AFR		1		1		1			1	1			1	1	1	1	1		
AUS				1						1									
CAN	1			1		1			1	1	1	1	1	1	1	1	1		
CHI																			
CSA	1			1		1			1	1		1	1	1	1	1	1		
EEU																			
FSU	1	1	1	1	1	1			1	1		1	1	1	1	1	1		
GBL																			
IND																			
JPN																			
MEA	1	1	1	1	1	1	1		1	1		1	1	1	1	1	1		
MEX															1				
ODA		1		1					1	1				1					
SKO																			
USA	1	1	1	1	1	1	1		1	1	1	1	1	1		1	1		
WEU															1		1		
UK			1												1	1			
MINRNW																			
IMPEXP																			

b)

~TradeLinks																			
GASNGA	AFR	AUS	CAN	CHI	CSA	EEU	FSU	GBL	IND	JPN	MEA	MEX	ODA	SKO	UK	USA	WEU	MINRNW	IMPEXP
AFR																	1		
AUS																			
CAN																1			
CHI														1					
CSA																			
EEU																	1		
FSU				1		1					1		1	1			1		
GBL																			
IND																			
JPN																			
MEA						1	1		1				1						
MEX																1			
ODA				1															
SKO																			
UK																	1		
USA			1									1							
WEU						1									1				
MINRNW																			
IMPEXP																			

Daniel Welsby:
Take out for non-TAPI runs

Daniel Welsby:
Constrain MEA_IND trade and no capacity start until 2025

Daniel Welsby:
Dependent on the Iran Pakistan (India) pipeline being built

Supplementary Figure 25. Trade links between TIAM-UCL regions for a) crude oil and b) natural gas via pipeline

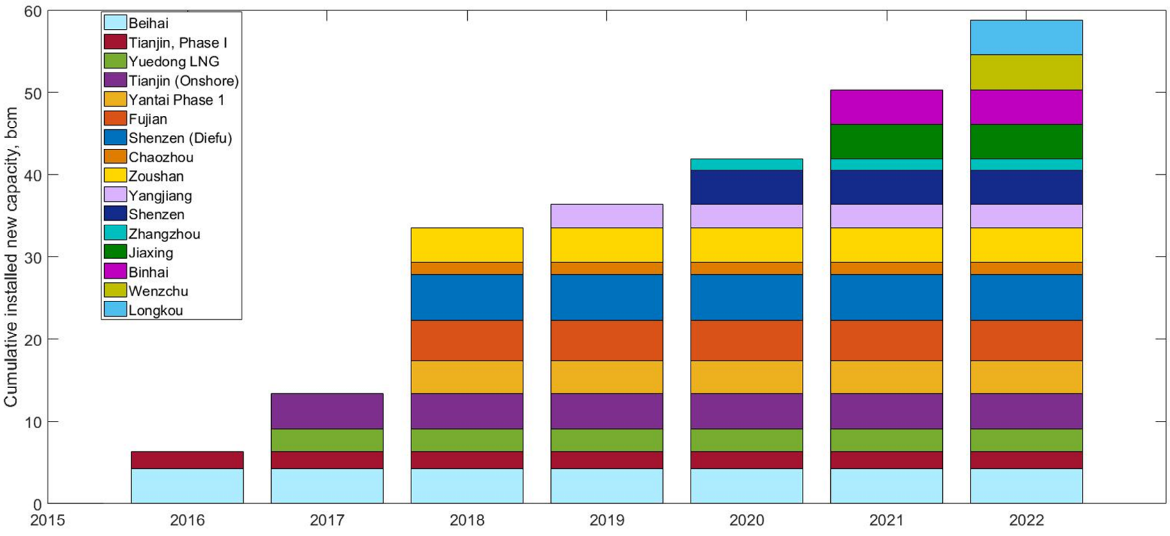

Supplementary Figure 26. Cumulative installed regasification capacity in China, 2016-2022. Source data are provided as a Source Data file. Source: IGU, 2019[4]

Supplementary Tables

Supplementary Table 1. Scenarios undertaken using the HDI criteria, under both SSP2 and SSP1 demands

Scenario name	Description
1.75C HDI LTX	HDI-based redistributive case under a 1.75°C target using a lower tax variant
1.75C HDI HTX	HDI-based redistributive case under a 1.75°C target using a higher tax variant
2C HDI LTX	HDI-based redistributive case under a 2°C target using a lower tax variant
2C HDI HTX	HDI-based redistributive case under a 2°C target using a higher tax variant
1.75C-A HDI LTX	HDI-based redistributive case under a 1.75°C target under SSP1 demands, using a lower tax variant
1.75C-A HDI HTX	HDI-based redistributive case under a 1.75°C target under SSP1 demands, using a higher tax variant
2C-A HDI LTX	HDI-based redistributive case under a 2°C target under SSP1 demands, using a lower tax variant
2C-A HDI HTX	HDI-based redistributive case under a 2°C target under SSP1 demands, using a higher tax variant

* Higher tax variants in 2030/50 are $196/$750 (Group 2) and $98/$375 (Group 1), while for lower tax variants the 2030/50 levels are $155/$375 (Group 2) and $78/$188 (Group 1).

Supplementary Table 2. Scenarios undertaken for two equity approaches, HDI and Benefits Accrued

Scenario name	Description
1.75C HDI LTX	HDI-based redistributive case under a 1.75°C target using a lower tax variant
1.75C HDI HTX	HDI-based redistributive case under a 1.75°C target using a higher tax variant
2C HDI LTX	HDI-based redistributive case under a 2°C target using a lower tax variant
2C HDI HTX	HDI-based redistributive case under a 2°C target using a higher tax variant
1.75C BEN LTX	Accrued benefit-based redistributive case under a 1.75°C target using a lower tax variant
1.75C BEN HTX	Accrued benefit-based redistributive case under a 1.75°C target using a higher tax variant
2C BEN LTX	Accrued benefit-based redistributive case under a 2°C target using a lower tax variant
2C BEN HTX	Accrued benefit-based redistributive case under a 2°C target using a higher tax variant

* Higher tax variants in 2030/50 are $196/$750 (Group 2) and $98/$375 (Group 1), while for lower tax variants the 2030/50 levels are $155/$375 (Group 2) and $78/$188 (Group 1).

Supplementary Table 3. Cost range for natural gas mining technologies in TIAM-UCL

Resource category	Minimum cost, $/boe	Minimum cost region	Maximum cost, $/boe*	Maximum cost region
Proved non-associated onshore conventional reserves (includes sour)	5	AFR_OPEC	40	USA
Proved non-associated offshore shallow conventional reserves	10	MEA_OPEC	33	AUS
Proved non-associated offshore deep conventional reserves	20	CSA_N	44	USA
Conventional non-associated reserve additions	10	FSU	48	ODA
Undiscovered non-associated conventional	24	FSU	61	MEX
Sour natural gas undeveloped	30	USA	57	MEA_P
Arctic	31	-	64	-
Shale gas	16	USA	130	MEA_N
Tight natural gas	19	USA/CAN	66	CSA_N
CBM	18	USA	63	CHI

* Natural gas costs here have been expressed in $/boe so they can be directly compared to the oil extraction costs in Table 1.3. Data from Welsby, 2020[2], McGlade, 2013[3].

Supplementary Table 4. Global availability of oil by resource category in TIAM-UCL

Oil category	Resource availability, EJ*	Share, %
Conventional	14842	52
Unconventional	13588	48
Global total	28400	

* Resource availability is the central value taken from McGlade, 2013[3].

Supplementary Table 5. Cost ranges for oil resources in TIAM-UCL (McGlade, 2013[3])

Resource category	Minimum cost, $/boe	Minimum cost region	Maximum cost, $/boe	Maximum cost region
Proved reserves	11	MEA_OPEC	47	CSA_N
Reserve addition	21	MEA_OPEC	68	CSA_N
New discoveries	16	MEA_OPEC	94	IND
Arctic	48	-	102	-
Bitumen (mining)	35	-	44	-
Bitumen (in-situ)	29	-	37	-
Ultra-heavy oil	29	-	37	-
Oil shale	39	-	83	-

* Sourced from McGlade, 2013[3].

Supplementary Table 6. Range of associated natural gas[1] investment and operational costs

Associated gas production field/region	CAPEX, $/MMBtu	OPEX, $/MMBtu	Region (Country)	Source
Bakken		0.31-0.67	United States (USA)	EIA (2016)[5]
Grand Rapids Bitumen	11.25	0.62	Canada (Canada)	AER (2018)[6]
Tengiz	2.40		Former Soviet Union (Kazakhstan)	Carbon Limits (2013)[7]
Middle East OPEC		0.8-1.3	Middle East OPEC countries	IPAA (2015)[2]
Utorogu	1.95		Africa (Nigeria)	OGJ (2016)[3]
Nigeria offshore		0.09-0.38	Africa (Nigeria)	Santley et al (2014)[8]
El Merk		0.27	Africa (Algeria)	Aissaoui (2016)[9]
Gassi Touil		0.60	Africa (Algeria)	Aissaoui (2016)[9]
Cantarell		0.18	Mexico (Mexico)	IMCO (2014)[4]
Ku-Maloob-Zaap		0.11	Mexico (Mexico)	IMCO (2014)[5]

* Sourced from Welsby, 2020[2].

Supplementary Table 7. Example of upstream transformation for mined oil sands into synthetic crude oil

Mining process	Output commodity	Primary transformation	Input commodity	Output commodity (efficiency)
Oil sands, mined bitumen	Oil sands	Production of synthetic oil from mined bitumen	Oil sands<br><br>UPS_Natural gas<br>UPS_Electricity<br>UPS_Heat (Steam)<br>UPS_Hydrogen<br>UPS_Biofuels	Crude oil (72%)<br>Heat<br>Flared and vented natural gas<br>CO_2<br>CH_4

---

[1] Associated natural gas is modelled differently, in the sense that the supply cost is the cost of separating and processing the natural gas to yield 'dry stripped gas'. This is because associated gas is based on oil extraction economics, and therefore a field level analysis was not possible. The TIAM-UCL energy systems model has been amended to include region-specific operational costs associated with separating and processing, as well as investment costs (CAPEX) for building new associated gas processing capacities if necessary.

[2] IPA Advisory (2015). Middle East Natural Gas: Is price administration a hurdle to gas development? August 2015, http://ipaadvisory.co.uk/media/1144/middle-east-gas-pricing-15_08_11.pdf (Accessed 20.02.20)

[3] Oil and Gas Journal article. *Nigerian operator lets contract for gas processing plant*. https://www.ogj.com/refining-processing/article/17250582/nigerian-operator-lets-contract-for-gas-processing-plant (Accessed 20.02.20)

[4] IMCO (2014). A changing world: Mexico facing the 21st century energy revolution. https://www.wilsoncenter.org/sites/default/files/media/documents/event/energy_mexico_imco.pdf (Accessed 20.02.20)

[5] Ibid.

Supplementary Table 8. Liquefaction investment costs by region and year in TIAM-UCL, $M/PJ

Year	AFR	AUS	CAN	CHI	CSA	EEU	FSU	IND	JPN	MEA	MEX	ODA	SKO	UK	USA	WEU
2006	6	6	6	6	6	6	6	6	6	6	6	6	6	6	6	6
2010	16	16	16	14	16	18	16	14	16	9	16	14	14	18	9	18
2015	10	20	16	14	16	18	16	14	16	9	16	14	14	18	9	18
2020	10	21	22	20	20	20	20	20	20	10	20	20	20	20	12	20
2025	25	21	22	20	20	20	20	20	20	10	20	20	20	20	12	20
2050	20	21	20	20	20	20	20	20	20	18	20	20	20	20	12	20

* Sourced from Songhurst, 2014; 2018[10,11]; adapted by Welsby, 2020[2].

Supplementary Table 9. Representative shipping costs for LNG between TIAM-UCL regions, $M/PJ

	AFR	AUS	CAN	CHI	CSA	EEU	FSU	IND	JPN	MEA	MEX	ODA	SKO	UK	USA	WEU
**AFR**	0	0	0	1.09	0.26	0	0	0.84	1.28	0.63	1.06	1.02	1.19	0.83	0	0.81
**AUS**	0	0	0	0.75	0	0	0	0.96	0.8	0	0	0.73	0.8	0	0	0
**CAN**	0	0	0	0.85	0	0	0	1.2	0.81	0	0.62	0.85	0.82	0	0	0
**CHI**	0	0	0	0	0	0	0	0	0	0	0	0	0	0	0	0
**CSA**	0	0	0	1.07	0	0	0	0.97	1.14	0.91	0.78	1.15	1.12	0.81	0	0.8
**EEU**	0	0	0	0	0	0	0	0	0	0	0	0	0	0	0	0
**FSU**	0	0	0	0.72	0	0	0	1.39	0.59	0	0	1.38	0.66	0.81	0	0.8
**IND**	0	0	0	0	0	0	0	0	0	0	0	0	0	0	0	0
**JPN**	0	0	0	0	0	0	0	0	0	0	0	0	0	0	0	0
**MEA**	0	0	0	0.83	0.81	1.02	0	0.6	0.94	0	1.28	0.74	0.92	0.97	0	0.85
**MEX**	0	0	0	0	0	0	0	0	0	0	0	0	0	0	0	0
**ODA**	0	0	0	0.66	0	0	0	0.71	0.73	0	0	0	0.69	0	0	0
**SKO**	0	0	0	0	0	0	0	0	0	0	0	0	0	0	0	0
**UK**	0	0	0	0	0	0	0	0	0	0	0	0	0	0	0	0
**USA**	0	0	0	1.09	0.84	0.91	0	1.21	1.11	0	0.72	1.1	1.09	0.85	0	0.9
**WEU**	0	0	0	0	0	0.63	0.64	0	0	0	0	0	0	0	0	0

* Sourced from Welsby, 2020[2].

Supplementary Table 10. Pipeline investment costs for a range of representative projects used in TIAM-UCL

Pipeline Name	Status	Investment Cost, $M/PJ	Investment Cost, $MM/km
Power of Siberia	Under-construction	10.38-23.02	5.27-11.67
Central Asia-China	Operational	3.51	3.99
TAPI	Proposed	6.66-8.33	7.11-8.89

Supplementary Table 11. a) User constraints for a range of oil mining technologies extraction processes and b) gas extraction processes in TIAM-UCL

a)

Mining technology	Growth coefficient	Decline coefficient
Conventional proved reserves	1.41 ($\sqrt{2}$)	0.93
Conventional reserve additions	1.41 ($\sqrt{2}$)	0.93
Conventional undiscovered	1.41 ($\sqrt{2}$)	0.93
Shale oil	1.07	0.8
Mined bitumen	1.07	0.8
In-situ bitumen	1.1	0.85

b)

Mining technology	Growth coefficient	Decline coefficient
Conventional proved reserves	1.41 ($\sqrt{2}$)	0.95[6]
Conventional reserve additions	1.41 ($\sqrt{2}$)	0.92
Conventional undiscovered	1.41 ($\sqrt{2}$)	0.92
Shale gas	1.27	0.83
Tight gas	1.12	0.83
Coal bed methane	1.12	0.83

Supplementary Table 12. Demand drivers used in TIAM-UCL scenarios

Category	Assumption	Values				Units
		2030	2050	2080	2100	
SSP2	Population	8.3	9.2	9.4	9.0	billion
	GDP	17	25	42	59	000 US$2005/cap
SSP1	Population	8.0	8.5	7.9	6.9	billion
	GDP	19	34	60	82	000 US$2005/cap

Supplementary Table 13. Bioenergy availability (central estimates)

Bioenergy category	Values by year, EJ potential				Source
	2030	2050	2080	2100	
Solid biomass	43	45	48	50	Daioglou et al. (2016)[12]
Energy crops	17	31	31	31	Marginal land availability and energy crop yields from Ricardo-AEA. (2017)[13]. Biomass Feedstock Availability. Final report for BEIS. Supply cost curves based on Hoogwijk et al. (2009)[14]
MSW	17	27	27	28	TIAM-ETSAP

[6] Regional variations are taken into account; therefore the numbers above may differ between regions. Additionally, the decline rate for conventional oil and gas fields will vary depending on the size of the field, the stage of decline, and the geological structure of the reservoirs (IEA, 2009). For example, larger fields generally exhibit slower rates of production decline, as shown with the value for conventional proved gas reserves which is taken from a representative decline parameter calculated from super-giant gas fields in the Former Soviet Union (e.g. Urengoy)[2].

Supplementary Table 14. Power generation costs and efficiency assumptions

Technology	CAPEX, $2005 /kW 2010	2020	2030	2040	2050	Efficiency, % 2010	2020	2030	2050
MSW combustion	5236	4862	4488		4114	23	27	30	33
Bioenergy combustion	2618	2431	2244		2057	28	31	34	37
Bioenergy combustion (dcn)	2880	2674	2468		2263	28	31	34	37
Bioenergy gasification	3080	2860	2640		2420	31	34	37	40
Bioenergy gasification (dcn)	3388	3146	2904		2662	31	34	37	40
Coal IGCC	2376					44	48	51	54
Coal super critical	1870					41	42	42	42
Coal ultra super critical	2277					46	48	49	50
Gas CCGT	990					56	59	61	63
Oil generation (dcn)	659					31	31	31	31
Oil generation	495					38	39	40	42
Coal IGCC w/CCS		3802	3564		3326		38	43	48
Coal USC w/CCS		3643	3416		3188		39	42	46
Gas CCGT w/CCS		1584	1485		1386		49	53	57
Geothermal shallow	2376	2310	2255	2200	2129				
Geothermal deep	3911	3644	3383	3108	2846				
Geothermal very deep		4978	4510	4015	3564				
Hydro dam	1650-6050	1623-5913	1595-5775	1568-5638	1540-5500				
Solar CSP	5850	3330	2700	2385	2070				
Solar PV	2587	923	633		376				
Tidal	6600	5500	4400		3432				
Offshore wind		2921	1749	1047	627				
Onshore wind		1314	969	715	527				
Nuclear Advanced LWR	3726	3524	3443		3240				
Storage	3300	1336	1034	688	472	80	80	80	80

Source: Fossil and CCS technologies (Ekins et al. (2017)[15]; Rubin et al. (2015)[16]); CCS is available from 2030, and can see capacity growth of 5% per annum. Power generation technologies have capture rates of 90%, which do not improve over time. Future solar PV and wind reductions based on BNEF estimates (unpublished), recent cost estimates based on IRENA[17]. The maximum build rate of new solar PV and wind capacity each year is set at 30% of existing capacity in line with recent solar PV build rates[18]. Range for hydro denotes different resource tranches and cost of exploitation. In the above table, 'dcn' denotes 'decentralised'.

Supplementary Table 15. a) BECCS technology costs and efficiency assumptions and b) other assumptions

a)

Technology Group	Technology Group	Efficiency %		Investment cost $/kW		Fix cost $/kW		Variable cost $/GJ	
	**Year**	**2030**	**2050**	**2030**	**2050**	**2030**	**2050**	**2030**	**2050**
Electricity	Energy Crop Combustion w CCS	26	31	3060	2618	175	131	6.9	6.6
	Energy Crop Gasification w CCS	29	34	3600	3080	206	173	1.7	1.7
	Solid Biomass Combustion w CCS	26	31	3060	2618	175	131	6.9	6.6
	Solid Biomass Gasification w CCS	29	34	3600	3080	206	154	1.7	1.7
Heat	Heat from biomass with CCS	63	65	1671	1419	189			
Hydrogen	Hydrogen from biomass gasification + CCS	42	44	4594	3516	322	246		
Advanced transport fuels	FT process w CCS using solid biomass	34	42	3630	2509	218	150	5.9	3.9
	FT process w CCS using energy crops	34	42	3630	2509	218	150	5.9	3.9

b)

Technology Group	Technology Group	Start time	Life yr	Availability / capacity factor	CO_2 Capture rate %	Build rate %
Electricity	Energy Crop Combustion w CCS	2030	25	0.85	90	5
	Energy Crop Gasification w CCS					
	Solid Biomass Combustion w CCS					
	Solid Biomass Gasification w CCS					
Heat	Heat from biomass with CCS	2030	30	0.6	90	3
Hydrogen	Hydrogen from biomass gasification + CCS	2030	30	0.9	90	5
Advanced transport fuels	FT process w CCS using solid biomass	2030	30	0.9	50	5
	FT process w CCS using energy crops					

Source: Butnar et al. 2020[19]

Supplementary Table 16. Hydrogen production technology costs and efficiency assumptions

Technology	Size	Fixed O&M costs (% capital costs)	Capital investment costs ($2005/GJ/y)	
			2025	2050
Coal gasification	Large	0.05	27	24
Gas SMR	Large	0.04	7	5
Gas SMR	Small / medium	0.04	17	14
Biomass gasification	Large	0.07	26	26
Biomass gasification	Medium	0.07	34	34
Biomass gasification	Small	0.07	43	43
Waste gasification	Medium	0.07	34	34
Electrolysis	Medium / small	0.05	27	17

Supplementary Table 17. LULUCF and non-CO_2 GHG emissions

Emissions	Values by year				Units	Source
	**2030**	**2050**	**2080**	**2100**		
CO_2 from LULUCF	2.5	1.2	-1.0	-1.5	$GtCO_2$	Trajectory sourced from SSP Public Database (Version 1.1) hosted by IIASA https://tntcat.iiasa.ac.at/SspDb/dsd?Action=htmlpage&page=welcome. Model-Scenario: IMAGE SSP2-26
CH_4	275,467	212,033	178,996	157,291	Kt	
N_2O	9297	8863	8320	7920	Kt	

Supplementary Table 18. List of regions and countries in the 16 region TIAM-UCL model

Region	Countries
Africa (AFR)	Algeria, Angola, Benin, Botswana, Burkina Faso, Burundi, Cameroon, Cape Verde, Central African Republic, Chad, Comoros, Congo, Côte d'Ivoire, Democratic Republic of the Congo, Djibouti, Egypt, Equatorial Guinea, Eritrea, Ethiopia, Gabon, Gambia, Ghana, Guinea, Guinea-Bissau, Kenya, Lesotho, Liberia, Libya, Madagascar, Malawi, Mali, Mauritania, Morocco, Mozambique, Namibia, Niger, Nigeria, Rwanda, Sao Tome and Principe, Senegal, Seychelles, Sierra Leone, Somalia, South Africa, South Sudan, Sudan, Swaziland, Togo, Tunisia, Uganda, United Republic of Tanzania, Zambia, Zimbabwe
Australia (AUS)	Australia, New Zealand
Canada (CAN)	Canada
Central and South America (CSA)	Anguilla, Antigua and Barbuda, Argentina, Aruba, Bahamas, Barbados, Belize, Bermuda, Bolivia, Brazil, Cayman Islands, Chile, Colombia, Costa Rica, Cuba, Dominica, Dominican Republic, Ecuador, El Salvador, Falkland Islands, Grenada, Guatemala, Guyana, Haiti, Honduras, Jamaica, Martinique, Netherlands Antilles, Nicaragua, Panama, Paraguay, Peru, Saint Kitts and Nevis, Saint Lucia, Saint Vincent and the Grenadines, Suriname, Trinidad and Tobago, Uruguay, Venezuela (Bolivarian Republic of)
China (CHI)	China, Taiwan, Tibet
Eastern Europe (EEU)	Bosnia and Herzegovina, Bulgaria, Croatia, Czech Republic, Hungary, Montenegro, Poland, Romania, Serbia, Slovakia, Slovenia, The former Yugoslav Republic of Macedonia
Former Soviet Union (FSU)	Armenia, Azerbaijan, Belarus, Estonia, Georgia, Kazakhstan, Kyrgyzstan, Latvia, Lithuania, Republic of Moldova, Russian Federation, Tajikistan, Turkmenistan, Ukraine, Uzbekistan
India (IND)	India
Japan (JAP)	Japan
Mexico (MEX)	Mexico
Middle-east (MEA)	Bahrain, Brunei Darussalam, Cyprus, Iran (Islamic Republic of), Israel, Jordan, Kuwait, Lebanon, Occupied Palestinian Territory, Oman, Qatar, Saudi Arabia, Syrian Arab Republic, Turkey, United Arab Emirates, Yemen
Other Developing Asia (ODA)	Afghanistan, American Samoa, Bangladesh, Bhutan, Cambodia, Democratic People's Republic of Korea, Fiji, French Polynesia, Indonesia, Kiribati, Lao People's Democratic Republic, Malaysia, Maldives, Mauritius, Mongolia, Myanmar, Nepal, New Caledonia, Pakistan, Papua New Guinea, Philippines, Samoa, Singapore, Solomon Islands, Sri Lanka, Thailand, Timor-Leste, Tonga, Vanuatu, Vietnam
South Korea (SKO)	Republic of Korea
United Kingdom (UK)	United Kingdom
USA (USA)	United States of America
Western Europe (WEU)	Albania, Andorra, Austria, Belgium, Denmark, Faroe Islands, Finland, France, Germany, Gibraltar, Greece, Greenland, Iceland, Ireland, Italy, Luxembourg, Malta, Monaco, Netherlands, Norway, Portugal, San Marino, Spain, Sweden, Switzerland, Vatican

Supplementary Notes

Supplementary Note 1. HDI-based redistributive scenarios

This Supplementary Notes describes additional results for the core scenarios presented in the main paper, based on HDI criteria for redistribution of fossil production. Results on fossil production are first described, showing change in production, compared to the optimal case, for the three main fossil fuels under 1.75°C and 2°C climate targets, and the absolute level of production under the scenarios.

Production of fossil fuels

Under the 1.75°C target, reductions in production of up to 59-62%, 59-62% and 87% are observed for gas, oil, and coal respectively in 2060, relative to current levels (Supplementary Figure 1c, f and i). The impact of the tax mechanism is to reduce overall production levels compared to the optimal case, with the higher tax variant producing a stronger shift towards increased production in LMHD regions. The shift is shown clearly in the left hand panel of Supplementary Figure 1, with gains in shares of annual production as positive values. For gas, significant production share gains for LMHD regions are only observed after 2040 (Supplementary Figure 1a-b), taking an additional 38/57% (2050/2060) in the high tax case, mainly at the expense of HHD regions. Under the low tax variant, increases in the same years are considerably lower, at less than 25%. The increases under the high tax variant require tax rates on HHD production of \$375/tCO_2 in 2050 and thereafter; in the lower tax case, they are \$188/tCO_2 in this period. Relative to the cost-optimal case, gas production levels in 2040 in LMHD are about the same, but then are 2-3 times higher in 2060.

For oil, the production taxes mean that LMHD regions increase market share by 2040 (Supplementary Figure 1d-e), but only maintain production levels observed in 2020, again at the expense of producers in HHD regions. The level is slightly higher under the higher tax case in 2060, and slightly lower in the low tax case (compared to 2020). Coal is extremely sensitive to the tax levels imposed, due to its carbon intensity, and therefore the low level of coal still produced in 2040/60 switches to LMHD regions, which see no tax level.

Under the 2°C target, reductions in production are lower, at 48-54%, 51-60%, and 75-80% for gas, oil, and coal respectively in 2060, relative to current levels (Supplementary Figure 2c, f and i). For gas, the larger market size allows for marginally higher increases in production in LMHD regions in 2060, compared to under 1.75°C. The increase in share however is lower, with HHD regions retaining higher market share (Supplementary Figure 2a-b). For oil, a similar pattern is observed, although the market share for LMHD group is again lower (as is the case for gas) due to the higher global production levels and HHD share (Supplementary Figure 2d-e).

Costs of redistribution

This section describes some of the additional detail on the cost results presented in the paper. Supplementary Figure 3 shows the breakdown in cost type for the high tax variant for the three HDI groups, under 1.75°C and 2°C targets, relative to the cost-optimal cases. For the LMHD group (left hand panel), all benefit from negative trade costs (export revenues greater than import revenues). Interpretation of this cost element is important, with estimates based on the physical flow of commodities between regions, multiplied by the shadow price of the commodity. The shadow price, which for example for oil, reflects the increase cost to the system of supplying an additional unit of demand for a barrel of oil. Shadow prices can be influenced by other constraints in the model so they should not be interpreted as equivalent to future oil prices. Trade gains in LMHD are counteracted to some extent by the cost of increasing production (based on new capital and annual O&M costs). However, in the main, the LMHD group see a net gain, albeit not a very large one in most cases.

The HHD group (middle panel) that includes the large producers of the Middle East and Russia (and other former soviet states) starts with net gains but by 2040, as the tax level increases, reduces production and moves toward having an energy trade deficit. Note that post 2040, some costs of production are negative as these countries have lower production levels than in the optimal case; some costs remain however for ongoing production, including fixed O&M costs and ongoing

payments on capital investment. Finally, the VHHD group have such high tax levels applied that they move primarily to import, with limited ongoing production.

Supplementary Note 2. HDI-based redistributive scenarios based on SSP1 demands

The results described in this section consist of the same scenario set as in the previous section except they use SSP1 demand drivers (Supplementary Table 1)). The motivation for these sensitivities is to explore redistribution when demand for fossil fuels is lower, recognising that this a key uncertainty in the modelling. SSP1 characterises a future of green growth with high resource efficiency, sustainable production methods and investment in human development. Economic growth is modestly increased, with a focus placed on environmentally benign technologies and energy efficiency. In SSP1, the global population rises to 6.9 bn people in 2100, compared to 9.0 bn in SSP2; in SSP1, global GDP rises to $566 tn by 2100, compared with $539 tn in SSP2. TIAM-UCL has been calibrated so that final energy consumption falls within the plume of results from the IAMs for SSP1[20]. This scenario approach was used in a recent TIAM-UCL analyses, published by UK Energy Research Centre (UKERC)[21].

Production of fossil fuels under the SSP1 demand cases show a stronger decline from 2020 levels. Under a 1.75°C target, 2060 levels reduce by 60-67% for gas, 69-73% for oil, and 91% by coal, compared to 2020. Under SSP2, the equivalent values are 59-62%, 59-62% and 87%. The largest difference under the SSP1 case is for oil, as observed in Supplementary Figure 4b, resulting in lower LMHD production levels in 2060 than observed in SSP2 cases. 2040 levels are less impacted, as it is only after this period that SSP1 / SSP2 demand levels significantly diverge. They therefore continue to produce at a level consistent with that seen in 2020.

LMHD gas production in the SSP1 scenario (Supplementary Figure 4a) is similar to that observed under the SSP2 case, with any overall reductions (compared to the SSP2 case) mainly impacting the taxed region groups (HHD and VHHD). Gas production remains at similar levels in 2040 as observed for 2020, but does increase by 2060, particularly under the high tax case. As shown in Supplementary Figure 5, the relative gain in share in LMHD, and losses for the other groups are much greater in 2060 than in the SSP2 cases.

The pattern in the 2°C cases (in Supplementary Figure 6) is similar to that described above. The main difference is that both LMHD and HHD groups have slightly higher production levels, due the larger market under this target. The relative changes in global production shares compared to the optimal (SSP1) scenario are shown in Supplementary Figure 7.

Costs under the SSP1 cases show a similar pattern to those observed under the core SSP2 cases under both climate targets (Supplementary Figure 8). The main difference is the absolute level of redistribution is lower, due to the lower levels of production particularly in the longer term. This is particularly evident under the higher tax variant (b,f) where the change in net costs, and cost reduction for the LMHD group are much lower.

Supplementary Note 3. Accrued benefit-based redistributive scenarios

For comparison to the HDI-based equity criteria used in the paper, we also explored production redistribution using an alternative equity approach. Countries were grouped into three categories based on the accrued rents per capita from fossil fuel production since 1970. The time series for rents, defined as the difference between the value of fossil fuel production at world prices and total costs of production, are expressed in terms of % GDP, and are sourced from the World Bank World Development Indicators set.[7] Rent levels are estimated using GDP values, which are then divided through by the population of the country in each time series year. This per capita metric is then

[7] Data are sourced from https://databank.worldbank.org/, and include the following indicators – i) Oil rents (% of GDP) (NY.GDP.PETR.RT.ZS), Natural gas rents (% of GDP) NY.GDP.NGAS.RT.ZS and iii) Coal rents (% of GDP) NY.GDP.COAL.RT.ZS

summed up over time, to provide a cumulative per capita rent metric. The per capita metric was chosen as opposed to absolute cumulative rent levels to reflect level of benefit spread across the population in a given year. This choice of per capita metric changes the order of regions, as shown in Supplementary Figure 9, with Canada (CAN) and Australia (AUS) move up the ranking (right hand figure), to second and third.

In Supplementary Figure 9b, we determined three categories based on the ranking – i) high benefit (MEA (Middle East), CAN, AUS, FSU (Former Soviet states), USA), ii) medium benefit (MEX (Mexico), UK, CSA (Central and South America), AFR (Africa), WEU (Western Europe)) and iii) low benefit (CHI (China), ODA (Other Developing Asia, EEU (Eastern Europe), IND (India), JPN (Japan), SKO (South Korea)). The same set of differentiated carbon-based production taxes used for the HDI analysis were then applied to these three groups, with the high benefit group being subject to the highest tax, the medium benefit group subject to the lower tax, and the low benefit group not subject to any tax. Compared to the HDI groupings, there are some interesting differences. Two of the largest producing regions, FSU and MEA, are now in the higher tax group (while previously were in the lower tax group, HDD). AFR is now subject to a production tax, albeit the lower tax level as part of the medium benefit group. Using the HDI criteria, AFR was not subject to any tax, being in the low development group (LMHD).

There are problems with this type of criteria, as with any chosen criteria. The arguments are well rehearsed by Caney[22] in his consideration of Extraction and Benefit criteria for allocation of production rights. Issues of debate include intergenerational benefit (benefits in the past may be less relevant to current situation), and who the beneficiaries were in the past (did the wider population benefit, or was it elites or corporations). Nevertheless, it is an interesting basis for criteria, providing an interesting comparison to the core criteria used in the paper, based on HDI. These issues are further discussed in Supplementary Note 4.

The same scenarios were undertaken for the accrued benefit criteria groupings, as for the HDI criteria groupings (Supplementary Table 2). The production levels for both types of equity criteria under a 1.75°C and 2°C target level are shown in Supplementary Figures 10 and 12 respectively, while the change of in the share of production relative to the optimal case shown in Supplementary Figures 11 and 13. The accrued benefit scenarios are in the left hand panel (yellow-green-blue colours) while, for comparison, the HDI-based scenarios are in the right hand panel.

The primary difference with the regional groupings under the accrued benefit criteria is that the largest producers (HBN) incur the highest tax rate – and therefore the largest impact. This differs from the HDI criteria, where the large producers of the Middle East and former Soviet states where in the middle HDD group, incurring the lower production tax.

Under a 1.75°C target, by 2040 in both tax variants, the HBN group is producing less gas in 2040 than observed for the optimal case in 2060. In the 2°C case, the absolute levels of production under the tax cases are similar by 2040 and 2060, although the relative reductions in their share (Supplementary Figure 13a-b) are higher due to the larger production levels observed in the optimal case (Supplementary Figure 12a). The MBN group's level of gas production (green bar) is not strongly affected in either the 1.75°C and 2°C scenario; despite the production tax incurred, its production holds up due the large reductions incurred by the HBN group. The largest increase is seen for LBN, which includes China and other developing Asia, particularly under the 2°C scenario where the overall global production levels are higher.

For oil, both LBN and MBN increase production levels in 2040 and 2060 under redistributive cases, with positive values seen in Supplementary Figures 11e-f and 13e-f (blue and green colour bars). This reflects more flexibility in the MBN group to respond, given the prominent oil producers in that group such as CSA and Africa. For coal, 2040/2060 levels are low – but shift entirely to the group which does not incur a tax, that is LBN.

The resulting system costs under both climate targets are shown in Supplementary Figure 14. Under the 1.75°C case, only the MBN group benefits (as shown by negative costs values relative to the optimal case) through additional oil production and resulting trade (shown in e-f); interestingly, the LBN group see costs increases. Despite an increase in LBN production, much of this will be used domestically in large Asian economies and not exported for revenues. The higher cost of these

resource means that this groups sees overall cost increases (blue colour bars in a-b). In the 2°C case, a similar pattern is observed, although additional costs compared to the optimal case increase more due to the higher levels of production in this scenario (as shown in Supplementary Figure 12).

Supplementary Note 4. Equity criteria for the reallocation of fossil fuel production

Two sets of equity criteria have been used in this paper as the basis for implementing a differentiated production tax on fossil fuel production across different regions; i) the current level of development, as measured by the Human Development Index (HDI), and ii) the level of accrued benefits from past production.

The first HDI-based criterion is applied based on the idea of 'Common but Differentiated Responsibilities and Respective Capabilities' used in the United Nations Framework Convention on Climate Change (UNFCCC)[22]. A low HDI value here is used as a means of identifying those countries that have a greater need for development. Countries in this category that also hold fossil fuel reserves, would under an HDI-based fossil fuel production redistribution mechanism, have a reduced 'responsibility' to forgo the income that they could receive from producing their reserves, and greater development need to use that income to fund development. Hence in our modelling, the tax incentive is used to shift production towards those countries that have reserves, and have a lower HDI.

Whilst there is a strong causal relationship between human development and energy consumption, the correlation between levels of human development and levels of fossil fuel production is weak. It is evident that some countries that hold fossil fuel reserves have been able to use export revenues from those reserves to drive development e.g. Norway, and a number of Middle East countries; however, many other countries with fossil fuel reserves have fallen to the resource curse[23]. Conversely, while there are countries without fossil fuel reserves that have achieved high development levels, some low HDI countries also have no fossil fuel reserves.

This weak correlation between HDI levels and access to fossil fuel reserves has at least two potentially problematic implications for any practical application of this kind of criterion. First, some low HDI countries are not fossil fuel producers, but importers. This means that an HDI-based redistribution of fossil fuel production would not benefit all low HDI countries. It would clearly not benefit those low HDI countries that do not have fossil fuel reserves. In fact, if the effect of the measure were to raise the costs of fossil fuel imports for such countries, then it could further disadvantage them.

Second, the use of this criterion is based on the premise that future production will provide an export revenue stream that low HDI countries will use to bring about positive and equitable society-wide development. However, this premise is not straightforwardly axiomatic. While there is no direct equivalent to fossil fuel production in terms of the rent and foreign exchange it can generate, fossil fuels production is not the only possible source of development. Other alternatives to development might be available that could reduce the need for future fossil fuel extraction and imports; indeed a fossil-based strategy could deter other more sustainable forms of development. Positive benefits to countries from fossil fuels extraction in the past are also no guide to future benefits; the sector is subject to large uncertainties (climate policy, alternative sources of energy) that will limit future gains as revenues decline. Furthermore, there is no guarantee that sustained or increased production would deliver development goals, and whether such gains would be equitably spread across a given country.

The current level of production by model regions in the different HDI groupings is shown in Supplementary Figure 15. Unlike in the accrued benefit case, some key large producers also feature strongly in the middle group that incurs the reduced tax rate, as opposed to all in the high tax group. This includes China for coal, and Middle East (MEA) and Former Soviet States (FSU) for oil and gas. At the aggregated regional scale, the LMHD group are comparably much smaller producers, although have some large single country producers. This contrasts to the accrued benefit case, where the largest producers are all in the group that incurs the highest carbon tax (see Supplementary Figure 9b).

As described in Supplementary Note 3, the second criterion featured in this paper concerns the benefits accrued since 1970, as measured by fossil fuel rents. The idea here is that those countries that have benefited most in the past should allow for other producers to transition away from fossil fuels at a slower pace. As with the HDI criterion, there are issues with its use. Historical cumulative benefit does not automatically mean that a specific country is in a strong position to transition away from fossil fuels. In fact, their dependency on the sector may be strongly entrenched, with limited economic diversification, making a managed exit from such activities challenging e.g. Russia. Furthermore, large accrued benefits do not necessarily mean high levels of equitable development, which again depends on how rents have been distributed across society.

Related to the above points, a third criterion suggested by Caney[22] (but not taken forward in this paper) concerns the ability of countries to develop based on non-fossil fuel alternatives should be considered, such that those countries with limited alternative development routes have greater opportunity to produce their fossil fuel resources. However, this is problematic where there is strong dependency on the sector for fiscal revenues. For example, Figure 2 in a recent paper by Muttitt and Kartha[24] shows that some African countries have a high dependency on oil revenues, as well low capacity to transition (as measured by GDP per capita). For such countries, transitioning away from fossil fuels will be particularly challenging. However, not all countries with high exposure to fossil fuels have low development, as seen in the case of Russia. If it was established as a general principle that high dependence on fossil fuels could translate into an argument for delayed transition, it could create a perverse incentive whereby countries delay economic diversification or even rush to lock-in to fossil production. As such, this criterion has minimal association with either need or responsibility, and seems to us to be significantly weaker than the other two; hence we chose not to model it, but to focus on the HDI- and accrued benefits-based criteria.

Supplementary Note 5. Overview of the TIMES modelling framework

TIAM-UCL uses the TIMES (The Integrated MARKAL-EFOM System) framework, a framework developed to explore and assess how energy systems may change over time, particularly in response to different policies e.g. climate or renewable energy targets, and drivers of energy demand e.g. economic growth. It is a partial equilibrium model, so balances supply and demand within the energy system, finding the least-cost solution for an energy system that meets future demand for energy services, such as space heating and cooling, mobility, and industrial production output.

A key feature of the model is that it is constructed from the technology level up, meaning that many of the system components e.g. refineries, vehicles, power stations, can be represented, including their performance and costs. An advantage of TIMES is that is can assess the role of these many different components within an interconnected system, exploring trade-offs, and dependencies, and providing a consistent assessment of the system as a whole. TIMES is also known as an E3 model, meaning that it provides metrics on the physical energy system (technology capacity, system operation, energy use totals), on the economics of the system (investment requirements, energy costs), and on the environmental impacts (GHGs).

TIAM-UCL, as typical of a TIMES model, is made up of five key components, as shown in Supplementary Figures 16. The first stage is the definition of what the system looks like (model structure), including the sectors to be included, typically from primary production through conversion to end use, types of technologies, and available energy resources. A Reference Energy System links all of these components together, allowing for the flow of energy through the system. Structure definition also include the spatio-temporal resolution e.g. number of regions, the number of time periods, and the time horizon over which the model is going to be run.

Each part of the model system is populated with data assumptions, including technology costs and performance, resource availability and cost, the demands that the system needs to meet today and in the future, and the associated emissions of different fuel-technologies.

The model input data are then used to construct a linear programme (using GAMS code), whereby the rules of the system operation and evolution are defined based on a set of mathematical equations. The linear programme is then solved to explore the least-cost energy system required to meet the

energy service demands in the future, subject to constraints. A large number of metrics result that describe the emerging energy system, in terms of the physical system (number of vehicles, power system capacity, total bioenergy use), the costs of that system, and the emissions resulting from the system's operation.

This section now focuses on the core equations used in the linear programme. This code is available on Github, with full documentation available on the ETSAP website[25] [8].

In simple terms, an optimisation model such as TIAM-UCL will –

- Minimise the objective function (total system costs)
- whilst satisfying the energy service demand requirements
- and respecting the system constraints

The key equations that set the rules of the model LP (linear programming) problem are summarised below:

- Objective function (EQ_OBJ). The function is to minimise total discounted system costs.

$$Min \quad \sum_{y} disc_{y} \left[\begin{array}{l} \sum_{p}\sum_{ts} \cdot varom_{y,p,ts} \cdot ACT_{y,p,ts} + \sum_{p} crf_{y,p} \cdot invcost_{y,p} \cdot NCAP_{y,p} + \sum_{p} fixom_{y,p} \cdot CAP_{y,p} \\ + \sum_{c}\sum_{ts} impprice_{y,c,ts} \cdot IMP_{y,c,ts} - \sum_{c}\sum_{ts} expprice_{y,c,ts} \cdot EXP_{y,c,ts} + \sum_{c}\sum_{p}\sum_{ts} flocost_{y,p,c,ts} \cdot FLO_{y,p,c,ts} \end{array} \right]$$

Where *disc* is the global discount rate, *varom* is the variable O&M costs associated with technology activity (*ACT*), *invcost* is the capital expenditure associated with new investment (*NCAP*), discounted using the *crf* (capital recovery factor), *impprice* is the price of imports, multiplied by import level (*IMP*), *expprice* is the price of exports, multiplied by export level (*EXP*), and *flocost* is the cost of other domestic energy commodities (*FLO*).

Where index *y* is year, *p* is process (technology), *c* is energy commodity, and *ts* is time segment.

- Commodity balance (EQ(l)_COMBAL). This equation ensures that the production of a commodity is equal to it consumption, to balance commodity markets.

$$\sum_{p \in Production} \sum_{ts} FLO_{t,p,c,ts} + \sum_{ts} IMP_{t,c,ts} = \sum_{p \in Consumption} \sum_{ts} FLO_{t,p,c,ts} + \sum_{ts} EXP_{t,c,ts}$$

- Transformation equation (EQ_PTRANS). This establishes the relationship between an input commodity to a technology and an output commodity e.g. technology efficiency

$$\eta_{t,p,cin,cout,ts} \cdot FLO_{t,p,cin,ts} = FLO_{t,p,cout,ts}$$

Where η is the efficiency factor, and FLO_{cin} and FLO_{cout} represent the input and output commodities of a technology.

[8] The TIMES model code can be found on GitHub at https://github.com/etsap-TIMES/TIMES_model. Documentation can be found at https://iea-etsap.org/index.php/documentation

- Product allocation constraint (EQ(l)_INSHR/OUTSHR). Allows for the control of different commodity shares, where there are more than one input or output commodities into a technology.

$$\frac{FLO_{t,p,com,ts}}{\sum_{c \in cg} FLO_{t,p,c,ts}} \leq (=, \geq)\, floshar_{t,p,com,cg,ts,bd}$$

Where *floshar* defines the share of the single commodity (numerator) over the sum of commodities (denominator), or commodity group (index *cg*).

- Activity definition (EQ_ACTFLO). Activity of a technology is a function of the commodity flow, either of inputs but more typically outputs.

$$ACT_{t,p,ts} = FLO_{t,p,c,ts}$$

- Utilization constraint (EQ_CAPACT). Ensures that the activity of a technology is a function of its capacity

$$ACT_{t,p,ts} \leq \alpha_{t,p,ts} \cdot CAP_{t,p}$$

While the above equations constitute the key set used in the linear programme, a full listing can be found in Loulou et al. (2016)[25], in Table 24. These include specific equations that bound capacity, activity and commodity production, set the rules for the operation of storage technologies, or ensures capacity exceeds demand for a selected commodity in a given time period (often used to ensure a peak margin for electricity systems).

User defined equations can also be built to provide more control over the model operation. Most are built using the standard LHS form, where the left hand side of the equation includes the variables to be controlled, while the right hand side (RHS) set the rule e.g. must be greater than 10% of total generation (share) or less than 50 GW capacity (absolute). Other user constraints are more dynamic in nature e.g. growth constraints that set % changes on the preceding period levels. A number of these constraints are outlined in the next section.

Supplementary Note 6. Characterisation of the fossil fuels sector in TIAM-UCL

The fossil fuel upstream sector in TIAM-UCL incorporates the availability and costs of primary energy resources, all extraction processes, and any upgrading / processing required to produce and distribute energy products for use in end-use sectors. The representation therefore captures the full system, from getting the resource out of the ground to a form where it can be used in another downstream sector and / or traded to another region. In this section, we describe the following parts of the sector representation –

- Fossil resource extraction
- Primary transformation (upgrading and/or processing)
- Trade
- Specific constraints used in the sector

Fossil resource extraction

Primary extraction in TIAM-UCL is represented by a range of 'mining' processes, with the costs reflecting a range of factors including:

- Technology maturity (e.g. if a technology has been developed over time in a certain region, and experience has been accrued (learning-by-doing), then the technology can be relatively mature and potential cost reductions/efficiency gains can be experienced. For example, shale gas drilling experience was accrued over decades in the United States, both through learning-by-doing (e.g. multiple wells per drilling pad) and fiscal incentives (tax breaks) which reduced costs, and therefore US shale gas costs are lower than other regions.
- Technical difficulty (generally driven by the geology of the formation in question, i.e. the source rock). Higher mining costs are incurred for fossil resources which require additional stimulation to lead to economic flow rates, such as hydraulic fracturing for tight reservoirs of oil/gas
- Production flow rates, linked to the above technical difficulty. Additionally, various source rocks yield different production flow dynamics, e.g. across the lifetime of a well, production characteristics for conventional and unconventional gas vary. Conventional non-associated natural gas would generally exhibit slower growth rates up to peak production, a longer production plateau period, and slower rates of production decline. Unconventional shale gas wells would generally exhibit rapid production growth up to a maximum, a shorter plateau vis-à-vis conventional gas, and faster decline rates.

Coal

Primary coal resources in TIAM-UCL are split into two categories, utilizing data collected by Remme et. al.[26] -

- Brown coal (lignite): lower energy content, with average heating value ranging from 5.57-17 MJ/kg
- Hard coal (sub-bituminous, bituminous and anthracite): higher energy content, with average heating value ranging from 17.58-27.55 MJ/kg

As with oil and natural gas, the extraction technologies for coal split the resource base into cost tranches, in order to reflect (albeit simplistically), cost depletion dynamics. In short, as the more accessible and higher quality resources are depleted, the model must move to more expensive extraction of (potentially) harder to exploit resources. Coal is split into hard coal and brown coal, with the representative mining technologies for both categories split into three cost categories. The distribution of resources/reserves assigned to each cost category varies by region, and is influenced

by the proportion of the total resource base which can be considered reserves[9], with the remainder of resources split between the middle and highest cost categories.

Supplementary Figure 17 shows a cost depletion curve for global coal resources, and the corresponding global supply cost curve constructed from the cost depletion curve. Additionally, Supplementary Figure 18 shows a global supply cost curve broken down into the regions of TIAM-UCL.

Natural gas

The underlying availability and cost of natural gas in TIAM-UCL is disaggregated into the following geological categories:

- Non-associated conventional gas proved reserves
- Non-associated conventional gas reserve additions
- Non-associated conventional gas new discoveries
- Associated natural gas
- Arctic conventional natural gas resources
- Shale gas
- Coal bed methane
- Tight natural gas

As with oil, the disaggregation of natural gas in TIAM-UCL is based on McGlade[3]. This analysis has been extended in a forthcoming thesis by Welsby[2], with field-level assessments of resource availabilities and costs. Resource assessments were generally conducted at disaggregated field-/play-level, and then aggregated into the regions of TIAM-UCL using probability distributions, and taking into account any correlation between discrete estimates etc. These were then applied to depletion curves which were formed from a database of field-/play-level costs where possible. The database was then extended to fields for which costs were either not known (i.e. no publically available indication of field supply costs) or have not yet been developed. This means the representation of natural gas supply costs in TIAM-UCL is driven by statistically significant coefficients of field-/play-level supply costs, aggregated into a representative cost depletion curves.

Supplementary Figure 19 shows the central supply cost curves for conventional non-associated natural gas resources (a significant focus of the work by Welsby[2]) and an aggregated global supply cost curve for all natural gas resource categories. The aggregated global curves below were mainly derived from field-/play-level data and have been aggregated from the TIAM-UCL regions. This means that some of the supply cost outputs will be higher / lower than the more disaggregated curves which formed the basis for these global supply cost curves.

Additionally, Supplementary Figure 20 shows a) the regional breakdown of the resource distribution, and b) the supply cost with each resource category identified. For reference, none of the figures in this section include associated natural gas resources in the supply cost curves, as these are calculated separately, with resource availabilities calculated by McGlade[3] and Welsby[2], and an endogenous decision within the model of whether to produce the gas or flare/vent it.

On a regional level, supply cost curves are constructed using an approach developed in Welsby[2]. Supplementary Figure 21a shows the aggregated output distribution for combining play-level estimates of shale gas for the Central and South America region in TIAM-UCL, while Supplementary Figure 21b shows the depletion analysis using US shale play cost analogues and linear regression using the geological characteristics of individual plays in South America (e.g. shale reservoir depth, thickness, etc.). These are then combined to form a (central) supply cost curve, shown in Supplementary Figure 21c.

Primary extraction of natural gas is split by region and into the different geological categories mentioned previously. For each category, the supply cost curves shown above are then

[9] Reserves are defined as geologically proven with current technologies, and commercially viable to extract at current market prices/cost conditions.

disaggregated into a three-step supply cost curve, with the first 50% of the resource base in the lowest cost bracket, then the next 30% in the middle cost category, and finally the last 20% in the highest cost category. An example of this split is shown in Supplementary Figure 22 taken from the work conducted on field-level analysis of the drivers of natural gas supply costs undertaken by Welsby[2]. It shows the constructed supply cost curve for the Middle East OPEC region in TIAM-UCL, with the low, middle and high cost ranges, and the corresponding quantity of proved non-associated gas reserves which can be extracted at each cost. For reference, the higher end of the cost range for the Middle East OPEC region would be sour gas deposits in the UAE and Saudi Arabia, some of which lie at significant reservoir depths[10].

Supplementary Table 3 shows a cost range for some key natural gas mining technologies, generated using a field-level database and a linear regression model applied to geological parameters to generate cost depletion curves[2].

<u>Oil</u>

The representation of oil in TIAM-UCL is predominantly based on the work by McGlade[3], which focused on quantifying uncertainties in the outlook for oil and natural gas, and in particular their availability and costs. As with natural gas, oil is split into different geological categories, each with specific availabilities and supply cost dynamics:

- Conventional oil proved reserves
- Conventional oil reserve additions
- Conventional oil new discoveries
- Arctic oil
- Mined shale oil
- In-situ shale oil
- Light tight oil
- Mined oil sands
- In-situ oil sands (ultra-heavy oil)

The representation of uncertainty in TIAM-UCL for oil availability and costs differs between conventional and unconventional oil. For conventional oil, direct estimates of reserve and/or resource availability were taken from the literature and input into probability distributions, with corresponding assumptions on correlation between the estimates. For unconventional oil (e.g. mined bitumen), two parameters were assigned probability distributions: a range of estimates for original oil in-place (OOIP) and a range of estimates of a recovery factor (i.e. between 0 and 1, which determines the proportion of the in-place resource base which is technically recoverable). These two distributions were then combined using random repeated sampling (Monte Carlo simulations) to form regional estimates. The combination is the product of the OOIP and the recovery factor, repeated a large number of times to generate an aggregated distribution. These estimates of the resource base for each category of oil were then combined with cost depletion curves, mostly formed from IEA data on cost ranges, and used to generate supply cost curves. Supplementary Figure 23, from McGlade[3] shows a) a range of cost depletion curves and b) example supply cost curves for proved oil reserves, taking into account the inherent uncertainty in any volumetric estimates. In general, the depletion analysis for unconventional oil exhibits significantly more rapid cost escalation (compared to conventional oil) as the resource base is depleted. Additionally, Supplementary Table 4 shows the split of oil into each category.

The mining processes for oil and natural gas match the geological categories listed previously. Unconventional oil (tar sands and oil shale) has several more steps in the model to reflect the upgrading required to generate a barrel of crude oil (i.e. to get from bitumen/kerogen, to a barrel of synthetic crude oil). Supplementary Table 5 shows the range of costs in TIAM-UCL for the mining technologies in the upstream sector. Also included is the region in TIAM-UCL containing the minimum and maximum cost for each category. As with natural gas represented in Supplementary Figure 22),

[10] Despite the costs, which are far above the highly regulated pricing of many OPEC states in the Middle East, these fields have been developed in some countries to limit import dependency (e.g. UAE), and have therefore been included in the assessment of proved reserves.

the supply cost curve is split into three sections: the first 50% of the resource base considered the lowest cost, then the next 30%, and finally the most expensive oil representing the last 20% of the resource base.

Due to limited development outside of certain countries (e.g. Canada for bitumen production), costs have largely been applied homogenously across the relevant TIAM regions. Supplementary Figure 24 shows the global supply cost curve for oil in TIAM-UCL split by region (a) and resource category (b). It should also be noted that unconventional oil is split into three separate cost categories: variable O&M, fixed O&M, and an investment cost (i.e. capital cost). In order to incorporate these into a supply cost curve with conventional oil, a singular supply cost figure was required, therefore the O&M costs were summed, and then a per-unit investment cost was assigned to each category of unconventional oil (derived by dividing cumulative investment and cumulative production from each mining technology) which then yielded a supply cost figure.

Primary transformation (upgrading and/or processing)

In addition to differentiating primary energy resources, TIAM-UCL also accounts for the further upgrading and processing of energy commodities (e.g. heat inputs required for the production of crude oil from the oil sands mining process).

Coal

Coal requires far less upstream upgrading/processing than oil and gas. In general any impurities will result in lower energy content in its end-use, rather than being removed in the upstream as with oil and gas. One main exception is the production of coking coal from hard coal, which requires energy inputs and therefore incurs costs, losses, and emissions across the upstream sector. Coking coal, used predominantly in the production of iron and steel is represented in TIAM-UCL as a product of heating hard coal at very high temperatures (≥ 1000°C), leaving very high concentrations of carbon.[11]

Natural gas

Natural gas requires processing to ensure it is of pipeline / liquefaction quality. This involves removing any impurities which could undermine the integrity of transportation / further transformation infrastructure, such as hydrogen sulphide (H_2S)[12] and carbon dioxide (CO_2) corroding gas pipelines, or CO_2 in a liquefaction terminal which would freeze at a much higher temperature than methane liquefies, and therefore lead to blockages/system shutdown at the facility.

After the mining process in the model, natural gas then passes to an upstream process for the collection and processing of natural gas from the well-heads to gas processing plants. Regionalised operation and maintenance costs are associated with this processing technology, as well as historical capacities. Additionally, a distinction is made between the gathering and processing of non-associated conventional natural gas, and unconventional natural gas. This process (taking into account emissions intensities, efficiencies, and any required energy inputs) turns the output gas from the mining process into an energy commodity which can either be traded internationally, via pipeline or LNG, or can be used as ‘useful’ input downstream (in conversion or end use sectors).

Due to its large regional-scale and intensive data requirements, TIAM-UCL is not able to accurately reflect the techno-economic characteristics of smaller downstream distribution networks for natural gas (e.g. distribution networks in urban areas which transport gas to individual households for cooking and heating service demand) due to the granularity required to effectively model such networks. However, additional user constraints have been added in the model to prevent excessively large and

[11] World Coal Association website. https://www.worldcoal.org/coal/uses-coal/how-steel-produced (Accessed 22.02.20)
[12] Hydrogen sulphide is not explicitly modelled in TIAM-UCL, due to the fact it is not a greenhouse gas. However, as part of the cost database work described in 1.2.3, a binary variable was included in the linear regression to isolate the impact on field supply costs of the presence of hydrogen sulphide. This variable was found to be statistically significant. Therefore whilst the physical presence of H_2S is not modelled, TIAM does capture that natural gas with high concentrations of H_2S is generally more expensive to produce than ‘sweeter’ counterparts.

unrealistic uptake of downstream natural gas, particularly for the residential sector, in regions/sub-regions where this is highly unlikely, at least in the near-term.

Additionally, underlying capacities and new capacity costs have been added into TIAM-UCL for associated natural gas[2], in order to reflect the fact that whilst it is produced as a relatively low cost by-product of oil, it still requires infrastructure to be in place, and is therefore a key reason behind large-scale flaring and venting in some regions. Supplementary Table 6 shows a range of investment and O&M costs for associated natural gas projects, which have been incorporated in TIAM-UCL. These improvements led to a more accurate recalibration of actual production volumes of associated natural gas in the near-term.

<u>Oil</u>

Oil generally requires the most refining, upgrading and processing in the upstream sector prior to being sent further downstream (e.g. crude oil as a traded commodity, or derived naphtha as a feedstock into petrochemical production of plastics etc.). In particular, for some forms of unconventional oil, a huge operation is required to upgrade the oil to 'useful' forms of energy (e.g. crude oil), which requires large-scale investment in upgrading infrastructure and intensive energy inputs into the processes. For example, extra-heavy oil and bitumen oil require significant upgrading to reduce the viscosity of the oil from a tar-like liquid (hence the name tar-sands) to a less viscous compound which can be transported by pipeline. A significant part of the improvements made to the upstream sector of TIAM-UCL was to provide insights into the costs and availability of unconventional oil production[3]. These costs and material flows[13] can be separately assessed in the model prior to the processed product eventually being used downstream. These upstream processes to upgrade and process require energy inputs, which have a range of efficiencies and costs. This is important for ensuring cost and additional energy use, and emissions, are captured, beyond the energy use and emissions associated with the initial extraction phase. This is of particular importance for bitumen and extra-heavy oil, where the upgrading process can account for upwards of 50% of the production cost (i.e. generating synthetic crude).[14]

TIAM-UCL has a detailed representation of the use of upstream energy fuels, whereby an energy commodity output from the upstream sector requires energy commodity inputs to produce a useable commodity downstream. An example of this would be the use of natural gas in in-situ and mined oil sands production, whereby the gas is used to generate steam/heated water, which increases the temperature of the oil in the reservoir or separates the oil from the sand, increasing the viscosity which allows it to flow at sufficient rates[15]. Supplementary Table 7 shows a (simplified) section of the upstream for the production of crude oil from synthetic mined bitumen, as well as the upstream energy requirements (input commodities with subscript UPS_).

[13] Including externalities associated with production of oil and gas, such as fugitive emissions, flaring, emissions from the upgrading process, etc.

[14] For example, McGlade (2013, p. 124) identifies the difference in costs (driven by energy requirements to upgrade, and the initial complexity/efficiency of the original mining process) between in-situ and mined bitumen, with mined the upgrading costs of mined bitumen reaching over 50% ($22/bbl) of the total production cost ($40/bbl). It should be noted these figures do not include fiscal regime costs.

[15] Canadian Energy Regulator website. *Market Snapshot: Natural gas plays an important role in Alberta's oil sands.* https://www.cer-rec.gc.ca/nrg/ntgrtd/mrkt/snpsht/2017/04-03ntrlgslbrtlsnd-eng.html?=undefined&wbdisable=true (Accessed 23.02.20)

Energy trade

Once extracted and processed, fossil fuels can then be transported between regions. An underlying trade matrix is used to determine inter-regional trade flow opportunities. For flexible forms of transportation (i.e. by maritime transport), the number of trade links will be higher than more constrictive forms of trading energy commodities (e.g. by pipeline, which are not just restricted by cost but also by geopolitical and geographical constraints). Supplementary Figure 25a shows a representative trade matrix for crude oil, with the number of trade links (represented by the number 1) significantly higher due to the flexibility of ocean tankers over pipelines[16], with the significantly lower number of trade links for natural gas via pipeline shown in Supplementary Figure 25b. The comments in this figure reflect some of the main uncertainty in pipeline routes, with the several projects stalling over several years and with no final investment decision taken. Therefore, the decision whether to switch these trade links on/off rests with the user.

Coal

It is assumed in TIAM-UCL that only higher grade coal is traded; i.e. sub-bituminous, bituminous and anthracite. All trade flows for coal have been recalibrated in the model to ensure that 2015-2020 flows of coal around the world are consistent with historical data[27,28]. As with natural gas (and oil) discussed subsequently, the trade of coal incurs costs, namely for its transportation via international shipping or across land-borders (i.e. by rail). The transportation costs, as with natural gas and oil, are determined based on average shipping/train capacities and rental rates, and the distance between the regions. However, unlike natural gas which requires processing, transformation and transportation infrastructure (e.g. liquefaction plants and pipelines), coal can be more easily transported and therefore no investment costs are required.

Natural gas

Natural gas trade in TIAM-UCL is split between pipeline gas and liquefied natural gas (LNG). Both are constrained firstly by the underlying trade matrix shown above. Additionally, trade volumes and infrastructure have been calibrated to 2015/2020-2025, with under construction infrastructure (both pipeline and LNG) fixed to come online in the model by 2020/2025, depending on an estimated start-date[29,30]. For example, Supplementary Figure 26 shows under construction regasification capacity for China between 2016 and 2022, which is used to bound the build rates of trade infrastructure capacity.

- LNG

Liquefied natural gas trade in TIAM-UCL includes infrastructural parameters (liquefaction and regasification capacities, and build constraints) and cost parameters (CAPEX on new infrastructure, OPEX on the liquefaction/regasification process, and a shipping cost). Regionalised liquefaction costs have been included based on:

- Representative projects in each region, including the location of the liquefaction terminal and investment costs;
- Competition for E&P in recent years which led to real price inflation on projects built between 2010 and 2020[11];
- Whether the project was a brownfield extension or conversion (e.g. conversion of regasification (import) terminals in the United States into liquefaction (export) facilities), or green-field integrated project (e.g. Yamal LNG field and export facility development in Russia, and several projects in Australia including Gorgon, Ichthys (floating), and Wheatstone).

Supplementary Table 8 shows a range of investment costs for liquefaction terminals in TIAM-UCL, showing the cost inflation attributed to a large range of projects coming online at the same time, and the corresponding stabilisation of these costs. It clearly shows which regions have the potential to

[16] Crude oil is also traded via pipeline, notably in Russia/Central Asia into Europe and China, and in North America (Canada-USA, USA-Mexico, etc.). Therefore, the activity costs of these trade processes in TIAM-UCL are reflected by the operation and maintenance costs of pipelines rather than crude oil tankers.

take advantage of cost de-escalation for brownfield conversions/expansions[10], i.e. the USA and the Middle East, before (at least in this example) costs converge across regions for green-field investments. Additionally, the amount of capacity which can be converted / expanded under these lower costs has been limited to existing regasification capacity and/or a maximum upper limit based on proposed brownfield extensions.

LNG variable O&M (i.e. shipping) costs in TIAM-UCL are calculated based on a range of parameters[2,31]:

- Assumed distance between ports
- Average speed of tanker
- Average capacity of tanker; calculated based on average capacity of tankers which are assigned to fixed routes and/or average size of delivery
- Daily rental rate of tanker (the rental rate is highly volatile depending on available capacities in each basin and seasonal spikes in LNG demand[17], however for a long-term energy systems model a fixed figure is assumed based on McGlade et al. [31].
- Boil-off rate (i.e. efficiency of transportation process translated into losses of natural gas), which in turn is a function of journey time
- Loading/unloading time at each port

A database of LNG transportation costs has been developed[2], with representative average shipping costs between the TIAM-UCL regions used if more than one trade route is used. An example of these shipping costs between individual liquefaction and regasification terminals is shown in Supplementary Table 9 below. For reference, the exporters are in red, and the zeros reflect that a) there is no intra-regional trade in TIAM-UCL and b) some regions are exogenously determined (through the trade link matrix shown) not to be able to trade with each other.

User constraints for natural gas trade through LNG are employed for both the technology which covers overall export capacity (i.e. the liquefaction process technology) and the bilateral trade process itself. In short, this constrains the model from building new capacity too quickly and sending all of the potential output through a single trade link.

- Pipeline

For pipeline investment costs and capacity additions in the near-term, individual project costs and capacity have been added where appropriate (e.g. pipeline cost and maximum volume from Russia to China between 2015 and 2020 are based on the under-construction Power of Siberia pipeline, which is due to come online in 2020). Some examples of pipeline investment costs are shown in Supplementary Table 10, with each pipeline at different development stage[2]. However, other factors need to be taken into account including whether the pipeline has to cross challenging physical barriers (e.g. a sea or mountainous territory).

Additionally, a user constraint has been added as an upper bound on potential gas pipeline trade, with a similar functional form as the upstream constraints discussed in the section that follows. In short, it is assumed that the model can, at a maximum, double capacity across a ten year period for any trade route (e.g. add a new pipeline parallel to an existing one with the same capacity). Therefore, an exponential growth constraint is set in the following form shown in Equation 1:

$$PipeCap_{a\to b,t} \leq PipeCap_{a\to b,t-1} * PipeGro^{ts} + Seed_{r,t} \quad (1)$$

[17] Platts (2018). Could LNG shipping spot rates hit $250,000/day? https://blogs.platts.com/2018/10/19/lng-shipping-spot-rates-hit-250000-day/ (Accessed 20.02.20)

Where,

$PipeCap_{a \to b,t}$ = pipeline capacity between exporter *a* and importer *b*, in time period *t*

$PipeCap_{a \to b,t-1}$ = pipeline capacity between exporter *a* and importer *b*, in time period *t-1*, i.e. the preceding time period

$PipeGro^{ts}$ = pipeline growth coefficient, set at ~1.07 (i.e. allows a doubling of capacity over 10 years using the above formulation)

$Seed_{r,t}$ = seed value for region *r* and time-period *t*, which allows growth value to take hold if there is no historical trade link, or adds on to the growth constraint for absolute upper bound (i.e. slackness on the constraint). The seed value is added across the time-slice, rather than in each individual year. As with LNG, this is based on a maximum capacity addition across a time-slice.

For regions where volumes of trade are already well established and there is significant pipeline capacity in place, the seed value has been set to zero from 2020 (e.g. between the UK and Western Europe, and the USA and Canada). A seed value is included in these cases between 2006 and 2015 in case large increases in gas pipeline trade were in evidence, such as between the United States and Mexico after the expansion of shale gas in the Barnett shale play. In short, the seed value allows the model to expand trade up to the upper bounds which have been added for 2015 to calibrate natural gas trade to historical data. However, some trade links have a seed value from 2020 to allow the model to expand pipeline capacity over the growth coefficient alone. For example, the seed value for gas pipeline trade between the Former Soviet Union and China is bounded (upper) by the growth coefficient (1.07/a) and a seed value equivalent to the under-construction Power of Siberia pipeline operating between a minimum (70%) and maximum (90%) contracted quantity.

Oil

The trade of oil commodities is split into various different products, which are outputs of processing/transformation processes in the upstream: crude oil, heavy fuel oil, naphtha, natural gas liquids[18], and diesel. As with natural gas trade via LNG tankers, the variable cost of transporting oil via tankers is assumed to be a function of the distance between ports, the speed of the tanker, and the average capacity of a ship travelling from the exporter to the importer.

Key upstream constraints

Coal

Upstream constraints for coal extraction are not as widely applied for two main reasons. Firstly, the extraction of coal does not follow the same geological production profile of oil and gas extraction; i.e. the growth and decline of production profiles through time and different geological structures. As coal resources become depleted, the energy inputs required for extraction become more intensive, and increasingly risk intensive, and inefficient, production methods are employed (e.g. mountain top removal). Secondly, for decarbonisation scenarios meeting 2°C and below, coal is rapidly phased out of the energy mix. Traditionally, this decline has not been constrained (as in this paper) but if being considered further in ongoing model development.

[18] Longer chain hydrocarbons which are separated from the gas stream in processing plants, most of which form liquids at surface temperature and pressure, and includes ethane, propane, butane (Schlumberger, 2019; https://www.glossary.oilfield.slb.com/Terms/n/natural_gas_liquids.aspx, Accessed 17.02.20).

Oil and natural gas

There are a range of upstream user constraints which control the rate at which production of different categories of oil and gas can grow/decline. In short, these user constraints model the natural growth and decline of oil and natural gas. The predominant form of constraint is an exponential (constant) rate of growth/decline across a time-slice, using seed values if there is no residual (historical) productive capacity. Equation 2 shows the functional form of these growth (a) and decline (b) constraints, for extractive technologies with historical production, while (c) and (d) shows the same equations for technologies which are new and require a seed value.

$$Production_{i,t} \leq (Production_{i,t-1} * Growth^{ts}) + Seed_{i,t} \quad (2\ (a))$$

$$Production_{i,t} \geq (Production_{i,t-1} * Decline^{ts}) + Seed_{i,t} \quad (2\ (b))$$

$$Production_{i,t} \leq Seed_{i,t} * Growth^{ts} \quad (2\ (c))$$

$$Production_{i,t} \geq Seed_{i,t} * Decline^{ts} \quad (2\ (d))$$

Where,

$Production_i$ = production of oil/gas for mining process i

$Seed_i$ = seed value from which growth/decline coefficients are assigned to if no historical (i.e. *t-1*) volumes, and which is added to overall growth/decline constraint across each time-slice[19]

t = time period in the model (therefore *t-1* is the previous time-slice)

Growth = growth coefficient, where *Growth* ≥ 1

Decline = decline coefficient, where *Decline* ≤ 1

ts = time-slice length (i.e. *t – (t-1)*)

Therefore, for growth constraints, the production of an oil and/or gas mining technology in time slice *t* will be bounded (upper) by a maximum of production in time slice *t-1* multiplied by the growth coefficient to the power of the length of the time-slice. For decline constraints, the inverse applies: production in *t* will be bounded (lower) by a minimum of production in *t-1* multiplied by the decline coefficient to the power of the time-slice length. Supplementary Table 11 shows examples of the growth/decline coefficient parameters and seed values used in TIAM-UCL, for a range of oil (a) and gas (b) mining technologies.

For example, shale gas decline rates were calculated using well-level data from the United States (Marcellus). Over 950 shale gas wells were assessed over a 10 month period; the rate of decline for these wells, when aggregated, best fit a hyperbolic profile. The rate of change between the first year and last year was then calculated to give a constant annual rate of decline. In order to generate a decline parameter which fits the formulation of user constraints in TIAM-UCL (Equations 2 (a-d)), this annual decline rate was then re-calculated to an equivalent rate of decline but as a constant exponential rate of decline. For the shale gas wells examined, this translates as a decline coefficient of ~ 0.83, which when raised to the power of 5 (assuming a five year time-slice), gives a maximum rate of decline of ~ 60% between *t-1* and *t*. Additional constraints have been input as a proxy for controlling the expansion of associated natural gas. Whilst the production itself is a function of oil extraction (and oil economics), the infrastructural issues surrounding associated gas utilisation require some degree of user constraint. Therefore, an upstream constraint is placed on the speed at which associated gas processing and separation capacity can be added.

[19] For decline user constraints, the seed value is negative, therefore any addition is actually a subtraction to the production level relative to *t-1*

Supplementary Note 7. TIAM-UCL core assumptions and region definition

This supplementary information describes some of the core assumptions used in this analysis, and the model's region definitions, listed in Supplementary Table 18. The same model assumptions were used in this recent report by Pye et al.[32].

Demand drivers

For most of the scenarios assessed, population and economic growth drivers are based on the SSP2 'Middle of the Road' scenario narrative. These drivers are used to construct the energy service demands across different sectors. Some adjustments have been made to energy service demands to ensure final energy demand globally falls within the SSP2 marker model (MESSAGE) range. As SSPs are independent of climate ambition, defining the socio-economic backdrop that a given climate ambition has to be achieved within, the demands for SSP2 in TIAM have been tuned to match the marker model's base / reference SSP2 run with no climate constraints[20]. A similar approach has been applied to the SSP1 'Sustainability' narrative. Region specific values are used but global values are shown in Supplementary Table 12.

Bioenergy characterisation and availability

Bioenergy is characterised into first and second generation fuels. First generation fuels are represented as bioliquids (bioethanol and biodiesel from crops which might compete with food crops for land) and biomethane (gas captured from controlled landfill sites). Four types of second-generation bioenergy feedstock distinguished: i) Solid biomass (BIOSLD), comprising woody residues from forestry and agriculture; ii) Energy crops (BIOCRP), comprising second generation purposely grown energy crops (grassy and woody bioenergy crops); iii) Municipal waste (BIOBMU), comprises wastes produced by households, industry, hospitals and the tertiary sector that are collected by local authorities; and iv) Industrial waste (BIOBIN), Solid and liquid products (e.g. tyres, sulphite lyes (black liquor), animal materials/wastes), usually combusted directly in specialised plants to produce heat and/or power. For each of these fractions cost supply curves are specified within the model for each of the 16 regions, i.e. amount of biomass available at different costs in each region. Cost ranges for solid biomass range between 4-16 $/GJ, and for energy crops between 9-15 $/GJ, with zero cost for waste fractions. To avoid competition for land, energy crops are assumed to be grown only on marginal and degraded land. Importantly, only solid biomass and energy crops fractions can be used for BECCS, and traded between regions. Supplementary Table 13 lists the global potentials for each bioenergy type.

Conversion technology assumptions

A key input into the TIAM-UCL model is the assumptions on costs and performance of different conversion technologies, which produce low carbon vectors. This section provides an overview of the key assumptions for different technology groups. Supplementary Table 14 provides information regarding the power generation sector.

The negative emission technology used in the model for this analysis was BECCS, the assumptions for which can be found in Supplementary Table 15. The main 'brake' on this technology set is the bioenergy resource availability. Direct Air Capture (DAC) is not an option used in this analysis.

On the supply side, hydrogen production technologies are divided into three different scales: centralised large-scale, centralised medium and decentralised small-scale production. Large-scale plants are based on biomass, coal and gas with continuous production of hydrogen. These plants are available with and without CCS technology. Hydrogen produced from centralised plants are transported with two different transportation options: long-distance pipe line transportation (gaseous hydrogen) and liquefaction plus trucks (liquid hydrogen). Hydrogen production data is presented in Supplementary Table 16, and are based on the review by Dodds and McDowall, 2012[33].

Other key assumptions

- Climate module. The climate module is calibrated to MAGICC17[34], with values from the probability distribution selected to give a 60% chance that the temperature rise will remain below any level reported. The model can be run to allow temperature overshoot prior to 2100. Net negative accounting is also switched on, meaning that the system CO_2 level can go negative, through the implementation of NETs. For these scenarios in this paper, the climate module is used in conjunction with carbon budgets, with the non-CO2 trajectory constrained in line RCP2.6 assessments.
- LULUCF emissions. Land use and forestry (LULUCF) emissions of CO_2 are based on a fixed trajectory, using outputs from the IMAGE model, based on the RCP2.6 SSP2 case. They are net CO_2 emissions from deforestation, and reforestation in line with SSP2 RCP2.6 assumptions (Supplementary Table 17).
- Non-CO_2 GHGs. Some non-energy sector sources of CH_4 and N_2O are not explicitly represented in TIAM-UCL but rather included as an emissions trajectory based on the RCP database. Such sources include CH_4 from landfill and waste water, and agriculture (manure, rice paddies) and N_2O from industry (nitric and adipic acid) and agriculture. In this modelling, the RCP2.6 trajectory is used for climate ambition cases (Supplementary Table 17). Emissions of these gases from the energy sector (e.g. CH_4 leakage from natural gas extraction and transport) are capped under an overall constraint, which includes the sources above.
- Discount rate. The social discount rate used in the calculation of net present value (used as the basis for the objective function) is set at 3.5%.
- Base year. Model is calibrated based on 2005 IEA energy balances. Additional constraints have been introduced in the model to help represent the energy system in 2010 and 2015 - and to reflect projected emissions in 2020.

Additional documentation for TIAM-UCL can be found on the Integrated Assessment Modelling Consortium (IAMC) wiki at https://www.iamcdocumentation.eu/index.php/Model_Documentation_-_TIAM-UCL.

Supplementary References

1. IEA. World Energy Balances (2019 Edition). https://doi.org/10.5257/iea/web/2019 (2019).
2. Welsby, D. Modelling uncertainty in global gas resources and markets (forthcoming). (University College London, 2020).
3. McGlade, C. Uncertainties in the outlook for oil and gas. *Doctoral thesis, UCL (University College London).* (2013).
4. IGU. *Global natural gas insights*. https://www.igu.org/global-natural-gas-insights (2019).
5. EIA. *Trends in U.S. Oil and Natural Gas Upstream Costs*. https://www.eia.gov/analysis/studies/drilling/pdf/upstream.pdf (2016).
6. AER. *Natural Gas Supply Costs*. https://www.aer.ca/providing-information/data-and-reports/statistical-reports/st98/natural-gas/supply-costs (2018).
7. Carbon Limits. *Associated Petroleum Gas Flaring Study for Russia, Kazakhstan, Turkmenistan and Azerbaijan*. https://www.ebrd.com/downloads/sector/sei/ap-gas-flaring-study-final-report.pdf (2013).
8. Santley, D., Schlotterer, R. & Eberhard, A. *Harnessing African Natural Gas: A New Opportunity for Africa's Energy Agenda?* http://www.gsb.uct.ac.za/files/harnessingafricangasfinal.pdf (2014).
9. Aissaoui, A. *Algerian Gas: Troubling Trends, Troubled Policies*. https://www.oxfordenergy.org/wpcms/wp-content/uploads/2016/05/Algerian-Gas-Troubling-Trends-Troubled-Policies-NG-108.pdf?v=79cba1185463 (2016).
10. Songhurst, B. *LNG Plant Cost Reduction 2014–18*. https://www.oxfordenergy.org/wpcms/wp-content/uploads/2018/10/LNG-Plant-Cost-Reduction-2014–18-NG137.pdf?v=79cba1185463 (2018).
11. Songhurst, B. *LNG plant cost escalation*. https://www.oxfordenergy.org/wpcms/wp-content/uploads/2014/02/NG-83.pdf?v=79cba1185463 (2014).
12. Daioglou, V., Stehfest, E., Wicke, B., Faaij, A. & van Vuuren, D. P. Projections of the availability and cost of residues from agriculture and forestry. *GCB Bioenergy* (2016) doi:10.1111/gcbb.12285.
13. Ricardo Energy & Environment. Biomass Feedstock Availability, Final Report for BEIS. *Ref ED662421043 - Issue Number 3* (2017).
14. Hoogwijk, M., Faaij, A., de Vries, B. & Turkenburg, W. Exploration of regional and global cost-supply curves of biomass energy from short-rotation crops at abandoned cropland and rest land under four IPCC SRES land-use scenarios. *Biomass and Bioenergy* (2009) doi:10.1016/j.biombioe.2008.04.005.
15. Ekins, P. *et al. The role of CCS in meeting climate policy targets*. https://www.globalccsinstitute.com/resources/publications-reports-research/the-role-of-ccs-in-meeting-climate-policy-targets/ (2017).
16. Rubin, E. S., Davison, J. E. & Herzog, H. J. The cost of CO2 capture and storage. *Int. J. Greenh. Gas Control* (2015) doi:10.1016/j.ijggc.2015.05.018.
17. IRENA International Renewable Energy Agency. *Renewable Power Generation Costs in 2017*. *International Renewable Energy Agency* (2018). doi:10.1007/SpringerReference_7300.
18. IEA PVPS. *Trends 2018 in Photovoltaic applications*. *Survey Report of Selected IEA Countries between 1992 and 2014* https://iea-pvps.org/wp-content/uploads/2020/01/2018_iea-pvps_report_2018.pdf (2018).
19. Butnar, I., Broad, O., Solano Rodriguez, B. & Dodds, P. E. The role of bioenergy for global deep decarbonization: CO2 removal or low-carbon energy? *GCB Bioenergy* (2020) doi:10.1111/gcbb.12666.
20. Riahi, K. *et al.* The Shared Socioeconomic Pathways and their energy, land use, and greenhouse gas emissions implications: An overview. *Glob. Environ. Chang.* (2017) doi:10.1016/j.gloenvcha.2016.05.009.
21. Cronin, J., Pye, S., Price, J. & Butnar, I. *Biomass, afforestation and energy demand reduction: trade-offs in the route to decarbonisation*. https://ukerc.ac.uk/publications/afforestation-energy-demand/ (2020).
22. Caney, S. *Climate change, equity, and stranded assets*. *Oxfam America Research Backgrounder. Washington, DC: Oxfam America* https://s3.amazonaws.com/oxfam-us/www/static/media/files/climate_change_equity_and_stranded_assets_backgrounder.pdf (2016).
23. Lahn, G. & Stevens, P. *The curse of the one-size-fits-all fix: Re-evaluating what we know about extractives and economic development*. https://www.wider.unu.edu/publication/curse-

one-size-fits-all-fix (2017).
24. Muttitt, G. & Kartha, S. Equity, climate justice and fossil fuel extraction: principles for a managed phase out. *Clim. Policy* 1–19 (2020) doi:10.1080/14693062.2020.1763900.
25. Loulou, R., Lehtilä, A., Kanudia, A., Remme, U. & Goldstein, G. *Documentation for the TIMES Model: Part II*. https://iea-etsap.org/docs/Documentation_for_the_TIMES_Model-Part-II_July-2016.pdf (2016).
26. Remme, U., Blesl, M. & Fahl, U. *Global resources and energy trade: An overview for coal, natural gas, oil and uranium*. https://d-nb.info/1027991858/34 (2007).
27. BP. *BP statistical review of world energy*. https://www.bp.com/content/dam/bp/business-sites/en/global/corporate/pdfs/energy-economics/statistical-review/bp-stats-review-2019-full-report.pdf (2019).
28. IEA. *Coal Information 2019*. https://www.iea.org/reports/coal-information-2019 (2019).
29. IGU. *World LNG Report 2019*. https://www.igu.org/sites/default/files/node-news_item-field_file/IGU Annual Report 2019_23 loresfinal.pdf (2019).
30. IEA. *Natural gas Information 2019*. https://www.iea.org/reports/natural-gas-information-2019 (2019).
31. McGlade, C., Bradshaw, M., Anandarajah, G., Watson, J. & Ekins, P. *A Bridge to a Low-Carbon Future? Modelling the Long-Term Global Potential of Natural Gas*. https://ukerc.ac.uk/publications/gas-as-a-bridge/ (2014).
32. Pye, S., Price, J., Cronin, J., Butnar, I. & Welsby, D. *Modelling 'leadership - driven' scenarios of the global mitigation effort*. https://www.theccc.org.uk/publication/modelling-leadership-driven-scenarios-of-the-global-mitigation-effort-ucl-energy-institute/ (2019).
33. Dodds, P. & McDowall, W. *A review of hydrogen production technologies for energy system models*. https://www.lifestudy.ac.uk/bartlett/energy/research/themes/energy-systems/hydrogen/WP6_Dodds_Production.pdf (2012).
34. Meinshausen, M., Raper, S. C. B. & Wigley, T. M. L. Emulating coupled atmosphere-ocean and carbon cycle models with a simpler model, MAGICC6 - Part 1: Model description and calibration. *Atmos. Chem. Phys.* **11**, 1417–1456 (2011).
