# Supplementary material for: An equitable redistribution of unburnable carbon
Source: Nat Commun. 2020 Aug 7;11:3968. doi: 10.1038/s41467-020-17679-3 (PMC7414863; doi:10.1038/s41467-020-17679-3)
Supplement: Supplementary file 3 — Reporting Summary [file 41467_2020_17679_MOESM3_ESM.pdf]

nature research

Corresponding author(s): Steve Pye

Last updated by author(s): Jun 22, 2020

Reporting Summary

Nature Research wishes to improve the reproducibility of the work that we publish. This form provides structure for consistency and transparency in reporting. For further information on Nature Research policies, seeAuthors & Referees and theEditorial Policy Checklist .

Statistics

For all statistical analyses, confirm that the following items are present in the figure legend, table legend, main text, or Methods section.

n/a	Confirmed	
☒	☐	The exact sample size (*n*) for each experimental group/condition, given as a discrete number and unit of measurement
☒	☐	A statement on whether measurements were taken from distinct samples or whether the same sample was measured repeatedly
☒	☐	The statistical test(s) used AND whether they are one- or two-sided<br>*Only common tests should be described solely by name; describe more complex techniques in the Methods section.*
☒	☐	A description of all covariates tested
☒	☐	A description of any assumptions or corrections, such as tests of normality and adjustment for multiple comparisons
☒	☐	A full description of the statistical parameters including central tendency (e.g. means) or other basic estimates (e.g. regression coefficient) AND variation (e.g. standard deviation) or associated estimates of uncertainty (e.g. confidence intervals)
☒	☐	For null hypothesis testing, the test statistic (e.g. *F*, *t*, *r*) with confidence intervals, effect sizes, degrees of freedom and *P* value noted<br>*Give P values as exact values whenever suitable.*
☒	☐	For Bayesian analysis, information on the choice of priors and Markov chain Monte Carlo settings
☒	☐	For hierarchical and complex designs, identification of the appropriate level for tests and full reporting of outcomes
☒	☐	Estimates of effect sizes (e.g. Cohen's *d*, Pearson's *r*), indicating how they were calculated

Our web collection on statistics for biologists contains articles on many of the points above.

Software and code

Policy information about availability of computer code

Data collection	No software was used.
Data analysis	TIMES code v4.3.4 was used for the linear programming modelling, and is available via GitHub (https://github.com/etsap-TIMES/TIMES_model). Under an academic license, commercial programming language GAMS (v24.7) was used to write the code, and CPLEX v12.6.3.0 was the solver used to run the model.

For manuscripts utilizing custom algorithms or software that are central to the research but not yet described in published literature, software must be made available to editors/reviewers. We strongly encourage code deposition in a community repository (e.g. GitHub). See the Nature Research guidelines for submitting code & software for further information.

Data

Policy information about availability of data

All manuscripts must include a data availability statement. This statement should provide the following information, where applicable:

- Accession codes, unique identifiers, or web links for publicly available datasets
- A list of figures that have associated raw data
- A description of any restrictions on data availability

The results data and key source data (shown in the Supplementary Figures) are provided with this paper in an accompanying XLS. Other datasets used in the determination of the equity criteria include the Human Development Index (accessed here presented in this paper and supplementary information are available on request. http://hdr.undp.org/en/data) and fossil fuel rents data from the World Bank World Development Indicator set (accessed here https://databank.worldbank.org/). Other modelling input assumptions are available on reasonable request.

Field-specific reporting

Please select the one below that is the best fit for your research. If you are not sure, read the appropriate sections before making your selection.

☐ Life sciences ☒ Behavioural & social sciences ☐ Ecological, evolutionary & environmental sciences

For a reference copy of the document with all sections, see nature.com/documents/nr-reporting-summary-flat.pdf

Behavioural & social sciences study design

All studies must disclose on these points even when the disclosure is negative.

Study description	This is a quantitative modelling analysis, using a scenarios approach to explore fossil fuel production futures. It uses exclusively secondary data sources to parameterise the model, including current year energy balances, resource supply curves, and a range of technology studies. We have chosen this category in this form as we felt our disciplinary background of economic and engineering analysis best fit with social sciences.
Research sample	This study does not undertake any sampling.
Sampling strategy	There is none, as we do not undertake sampling.
Data collection	We use use published secondary data sources exclusively.
Timing	Not applicable, as we did not undertake sampling
Data exclusions	Not applicable, as we did not undertake sampling
Non-participation	Not applicable, as we did not undertake sampling
Randomization	Not applicable, as we did not undertake sampling

Reporting for specific materials, systems and methods

We require information from authors about some types of materials, experimental systems and methods used in many studies. Here, indicate whether each material, system or method listed is relevant to your study. If you are not sure if a list item applies to your research, read the appropriate section before selecting a response.

Materials & experimental systems

n/a	Involved in the study	
☒	☐	Antibodies
☒	☐	Eukaryotic cell lines
☒	☐	Palaeontology
☒	☐	Animals and other organisms
☒	☐	Human research participants
☒	☐	Clinical data

Methods

n/a	Involved in the study	
☒	☐	ChIP-seq
☒	☐	Flow cytometry
☒	☐	MRI-based neuroimaging
